# Associations between self-reported sleep patterns and health, cognition and amyloid measures: results from the Wisconsin Registry for Alzheimer’s Prevention

**DOI:** 10.1093/braincomms/fcad039

**Published:** 2023-02-24

**Authors:** Lianlian Du, Rebecca Langhough, Bruce P Hermann, Erin Jonaitis, Tobey J Betthauser, Karly Alex Cody, Kimberly Mueller, Megan Zuelsdorff, Nathaniel Chin, Gilda E Ennis, Barbara B Bendlin, Carey E Gleason, Bradley T Christian, David T Plante, Rick Chappell, Sterling C Johnson

**Affiliations:** Wisconsin Alzheimer’s Institute, University of Wisconsin-Madison School of Medicine and Public Health, Madison, WI 53726, USA; Department of Biostatistics and Medical Informatics, School of Medicine and Public Health, University of Wisconsin-Madison, Madison, WI 53792, USA; Wisconsin Alzheimer’s Institute, University of Wisconsin-Madison School of Medicine and Public Health, Madison, WI 53726, USA; Wisconsin Alzheimer’s Disease Research Center, University of Wisconsin-Madison School of Medicine and Public Health, Madison, WI 53792, USA; Department of Medicine, University of Wisconsin-Madison School of Medicine and Public Health, Madison, WI 53726, USA; Wisconsin Alzheimer’s Institute, University of Wisconsin-Madison School of Medicine and Public Health, Madison, WI 53726, USA; Department of Neurology, University of Wisconsin-Madison School of Medicine and Public Health, Madison, WI 53726, USA; Wisconsin Alzheimer’s Institute, University of Wisconsin-Madison School of Medicine and Public Health, Madison, WI 53726, USA; Wisconsin Alzheimer’s Disease Research Center, University of Wisconsin-Madison School of Medicine and Public Health, Madison, WI 53792, USA; Department of Medicine, University of Wisconsin-Madison School of Medicine and Public Health, Madison, WI 53726, USA; Wisconsin Alzheimer’s Institute, University of Wisconsin-Madison School of Medicine and Public Health, Madison, WI 53726, USA; Wisconsin Alzheimer’s Disease Research Center, University of Wisconsin-Madison School of Medicine and Public Health, Madison, WI 53792, USA; Department of Medicine, University of Wisconsin-Madison School of Medicine and Public Health, Madison, WI 53726, USA; Wisconsin Alzheimer’s Institute, University of Wisconsin-Madison School of Medicine and Public Health, Madison, WI 53726, USA; Wisconsin Alzheimer’s Disease Research Center, University of Wisconsin-Madison School of Medicine and Public Health, Madison, WI 53792, USA; Department of Medicine, University of Wisconsin-Madison School of Medicine and Public Health, Madison, WI 53726, USA; Wisconsin Alzheimer’s Institute, University of Wisconsin-Madison School of Medicine and Public Health, Madison, WI 53726, USA; Wisconsin Alzheimer’s Disease Research Center, University of Wisconsin-Madison School of Medicine and Public Health, Madison, WI 53792, USA; Department of Medicine, University of Wisconsin-Madison School of Medicine and Public Health, Madison, WI 53726, USA; Wisconsin Alzheimer’s Disease Research Center, University of Wisconsin-Madison School of Medicine and Public Health, Madison, WI 53792, USA; University of Wisconsin-Madison School of Nursing, Madison, WI 53705, USA; Wisconsin Alzheimer’s Disease Research Center, University of Wisconsin-Madison School of Medicine and Public Health, Madison, WI 53792, USA; Department of Medicine, University of Wisconsin-Madison School of Medicine and Public Health, Madison, WI 53726, USA; Wisconsin Alzheimer’s Disease Research Center, University of Wisconsin-Madison School of Medicine and Public Health, Madison, WI 53792, USA; Department of Medicine, University of Wisconsin-Madison School of Medicine and Public Health, Madison, WI 53726, USA; Wisconsin Alzheimer’s Institute, University of Wisconsin-Madison School of Medicine and Public Health, Madison, WI 53726, USA; Wisconsin Alzheimer’s Disease Research Center, University of Wisconsin-Madison School of Medicine and Public Health, Madison, WI 53792, USA; Department of Medicine, University of Wisconsin-Madison School of Medicine and Public Health, Madison, WI 53726, USA; Madison VA GRECC, William S. Middleton Memorial Hospital, Madison, WI 53705, USA; Wisconsin Alzheimer’s Institute, University of Wisconsin-Madison School of Medicine and Public Health, Madison, WI 53726, USA; Wisconsin Alzheimer’s Disease Research Center, University of Wisconsin-Madison School of Medicine and Public Health, Madison, WI 53792, USA; Madison VA GRECC, William S. Middleton Memorial Hospital, Madison, WI 53705, USA; Wisconsin Alzheimer’s Disease Research Center, University of Wisconsin-Madison School of Medicine and Public Health, Madison, WI 53792, USA; Department of Medical Physics, University of Wisconsin-Madison School of Medicine and Public Health, Madison, WI 53705, USA; Department of Psychiatry, University of Wisconsin-Madison School of Medicine and Public Health, Madison, WI 53719, USA; Department of Biostatistics and Medical Informatics, School of Medicine and Public Health, University of Wisconsin-Madison, Madison, WI 53792, USA; Wisconsin Alzheimer’s Disease Research Center, University of Wisconsin-Madison School of Medicine and Public Health, Madison, WI 53792, USA; Wisconsin Alzheimer’s Institute, University of Wisconsin-Madison School of Medicine and Public Health, Madison, WI 53726, USA; Wisconsin Alzheimer’s Disease Research Center, University of Wisconsin-Madison School of Medicine and Public Health, Madison, WI 53792, USA; Department of Medicine, University of Wisconsin-Madison School of Medicine and Public Health, Madison, WI 53726, USA; Madison VA GRECC, William S. Middleton Memorial Hospital, Madison, WI 53705, USA

**Keywords:** Alzheimer’s disease, sleep, cluster analysis, composite scores, amyloid beta

## Abstract

Previous studies suggest associations between self-reported sleep problems and poorer health, cognition, Alzheimer’s disease pathology and dementia-related outcomes. It is important to develop a deeper understanding of the relationship between these complications and sleep disturbance, a modifiable risk factor, in late midlife, a time when Alzheimer’s disease pathology may be accruing. The objectives of this study included application of unsupervised machine learning procedures to identify distinct subgroups of persons with problematic sleep and the association of these subgroups with concurrent measures of mental and physical health, cognition and PET-identified amyloid. Dementia-free participants from the Wisconsin Registry for Alzheimer’s Prevention (*n* = 619) completed sleep questionnaires including the Insomnia Severity Index, Epworth Sleepiness Scale and Medical Outcomes Study Sleep Scale. K-means clustering analysis identified discrete sleep problem groups who were then compared across concurrent health outcomes (e.g. depression, self-rated health and insulin resistance), cognitive composite indices including episodic memory and executive function and, in a subset, Pittsburgh Compound B PET imaging to assess amyloid burden. Significant omnibus tests (*P* < 0.05) were followed with pairwise comparisons. Mean (SD) sample baseline sleep assessment age was 62.6 (6.7). Cluster analysis identified three groups: healthy sleepers [*n* = 262 (42.3%)], intermediate sleepers [*n* = 229 (37.0%)] and poor sleepers [*n* = 128 (20.7%)]. All omnibus tests comparing demographics and health measures across sleep groups were significant except for age, sex and apolipoprotein E e4 carriers; the poor sleepers group was worse than one or both of the other groups on all other measures, including measures of depression, self-reported health and memory complaints. The poor sleepers group had higher average body mass index, waist–hip ratio and homeostatic model assessment of insulin resistance. After adjusting for covariates, the poor sleepers group also performed worse on all concurrent cognitive composites except working memory. There were no differences between sleep groups on PET-based measures of amyloid. Sensitivity analyses indicated that while different clustering approaches resulted in different group assignments for some (predominantly the intermediate group), between-group patterns in outcomes were consistent. In conclusion, distinct sleep characteristics groups were identified with a sizable minority (20.7%) exhibiting poor sleep characteristics, and this group also exhibited the poorest concurrent mental and physical health and cognition, indicating substantial multi-morbidity; sleep group was not associated with amyloid PET estimates. Precision-based management of sleep and related factors may provide an opportunity for early intervention that could serve to delay or prevent clinical impairment.

## Teaser

Du *et al*. identified sleep profile clusters based on self-reported sleep symptoms in late middle-aged adults. Sleep profiles were associated with differences in concurrent health outcomes, subjective reports of functioning and cognitive performance, but not PET amyloid estimates. Addressing sleep problems may benefit health and cognitive outcomes.

## Graphical Abstract

**Graphical Abstract:**
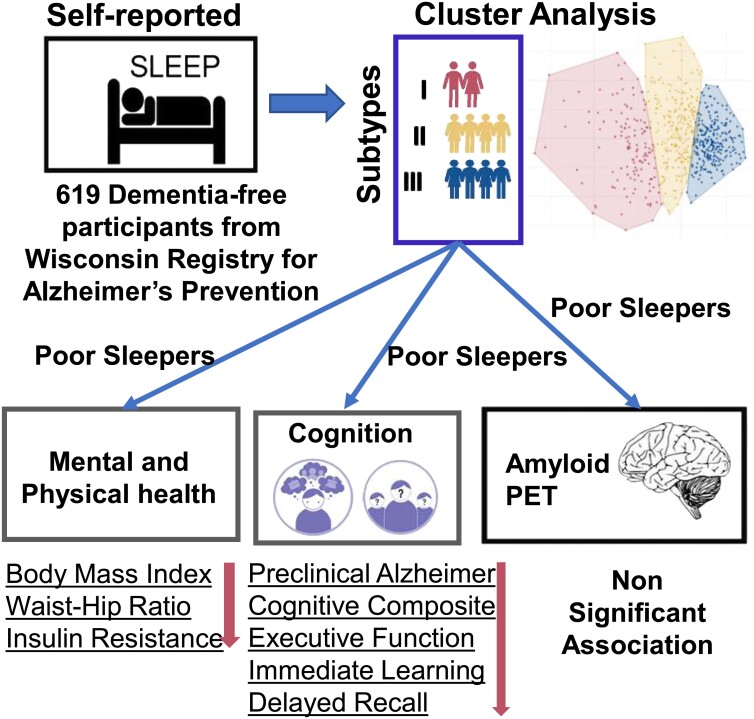

## Introduction

Alzheimer’s disease, the most common cause of dementia, accounts for an estimated 60–80% of prevalent cases.^1^ Approximately 40% of worldwide dementia cases are thought attributable to potentially modifiable risk factors,^2^ and emerging evidence suggests meaningful associations between various sleep disturbances (SDS) and dementia. The 2020 report of the Lancet Commission identified sleep as a putative risk factor for dementia^2^ with recent meta-analyses indicating that 60–70% of people with cognitive impairment or dementia have SDS^3^ including evidence that SDS are associated with a higher risk of all-cause dementia (RR 1.2; 95% CI 1.1–1.3)^4^ and clinically diagnosed Alzheimer’s disease (1.6, 1.3–1.9)^5^ compared to those with no SDS. Midlife insomnia and late-life terminal insomnia or long sleep duration have also been associated with a higher late-life dementia risk.^6^ Some investigations report that short and long sleep durations are associated with worse outcomes for older adults including greater amyloid-β burden and cognitive decline,^7^ but other studies^8-13^ do not find these associations.

Concerning in this literature are the variable definitions of SDS. Evidence regarding the growing burden of SDS may be limited predominantly to measures of sleep duration,^14-16^ or be limited to one or two measures of sleep,^6,7,9,17-20^ or may be defined broadly, or rely on different self-reported sleep characteristics such as short or long sleep duration, poor sleep quality, circadian rhythm abnormality, insomnia, or obstructive sleep apnoea. Another potential contributor to divergent findings is that ‘SDS’ often serves as an umbrella term encompassing different aspects of sleep dysfunction (e.g. insufficient quantity and poor quality) or related impairment in daytime functioning (e.g. daytime sleepiness). Therefore, the need exists to systematically examine the association between multiple indicators of SDS with meaningful outcome measures including cognition and Alzheimer’s disease risk. In addition, SDS are associated with a multitude of health indicators including poor self-rated health,^21^ depression,^22^ subjective memory problems,^23^ increased body mass index (BMI)^24^ and insulin resistance (IR).^25^ The presence, aggregation and association of these conditions, also viewed as modifiable risk factors, with identified sleep problems and cognitive and disease outcomes remain to be determined.

Methodologically, many studies have used the traditional *variable-centred* approach, investigating relationships between two or more variables in a given sample. Clarification of the nature of the relationship between multiple variables of interest is important, but uncertainty regarding how identified relationships may apply to all study participants potentially places limits on their clinical applicability. In contrast, *person-centred* analysis, an alternative approach, focusses on the identification of subgroups of individuals based on the dependent variable(s) of interest as well as the aggregation of comorbid variables inherent in each subgroup.^26^ Harnessing the heterogeneity inherent in study populations and phenotyping them into more homogeneous ‘groups’ offer to improve ecological validity and clinical utility and to advance understanding of the co-occurrence and interplay among multiple risk factors in these discrete groups.^27^

To the best of our knowledge based on literature reviews, this is the first report to understand the association between the individual part of sleep and Alzheimer’s disease risk in a group where people are unimpaired at baseline. Person-centred analyses have been used in sleep studies including with targeted clinical populations (e.g. apnoea^28^), individuals with comorbid psychiatric (major depression^29^) or other health conditions (COVID-19^30^) and among diverse sociodemographic groups (children,^31^ Caribbean Blacks^32^ and Australian patients^33^). Only four studies^19,34-36^ examined clusters of sleep problems in the general population; however, none of them examined the association between sleep, cognition and Alzheimer’s disease risk reflected in amyloid-β burden. In addition, three studies applied latent class analysis which only works on categorical variables^19,34,35^ and included only one sleep measurement.^19,35^ Most SDS were assessed using continuous variables; therefore, applying K-means and latent profile analysis (LPA) (two person-centred methods^37,38^) to sleep problems offers a better opportunity to identify the distribution of persons with heterogeneous patterns of sleep and their linked mental, physical and cognitive comorbidities.

In this study, we examine cross-sectional associations between sleep, health, cognition and positron emission tomography (PET) amyloid indicators from the Wisconsin Registry for Alzheimer’s Prevention (WRAP), a well-characterized midlife cohort at risk for Alzheimer’s disease. Our first aim is to identify discrete subgroups or clusters of participants with variable patterns of sleep efficiency using person-centred methods. The second aim is to determine to what degree the identified sleep clusters are associated with concurrent measures of cognition, mental and physical health assessed by self-report and objective measures, as well as PET-assessed amyloid. We hypothesize that discrete subgroups of increasingly severe sleep abnormality would be identified with linked risks of health and cognitive abnormalities and amyloid positivity.

## Methods

### Participants and study design

Participants were drawn from the WRAP, a longitudinal study designed to identify midlife factors associated with the development of Alzheimer’s disease.^39,40^ Enrolment of participants began in 2001, with the first follow-up visit occurring 2 to 4 years after the baseline visit and all additional visits occurring at 2-year intervals thereafter. WRAP participants were free of dementia at enrolment (mean age 54 years). All study procedures were approved by the University of Wisconsin School of Medicine and Public Health Institutional Review Board and are in concordance with the Declaration of Helsinki.

At each study visit, participants completed comprehensive neuropsychological assessment and multiple questionnaires related to a broad array of factors, including lifestyle, modifiable risk factors, medical history and memory functioning. Sleep measures were added in two stages to the WRAP assessment protocol. To be eligible for the primary analyses, participants needed to have completed the full set of sleep measures at least once and be free of dementia at time of sleep assessment (*n* = 619). To be eligible for secondary analyses, participants needed to have completed at least one of the sleep questionnaires described below and had completed a Pittsburgh Compound B (PiB) PET scan.

### Sleep assessment

The assessment protocol was expanded in 2012 to incorporate two self-report sleep measures [the Medical Outcomes Study Sleep Scale (MOS)^41^ and the Epworth Sleepiness Scale (ESS)^42^]; in 2014, the Insomnia Severity Index (ISI)^43^ was added to a specific visit, and in 2016, these assessments were included at all study visits.

#### The Sleep Scale from the Medical Outcomes Study

This scale comprises 12 questions about the past 4 weeks, from which eight scores were computed.^44^ The first question asks how long it takes to fall asleep, with possible responses in 15-min increments ranging from 1 = ‘0–15 minutes’ to 5 =‘More than 60 minutes’.^44^ The second question asks the average number of hours slept each night, which is entered freely.^44^ Responses to the remaining 10 questions are on a 6-point scale ranging from 1 = ‘all of the time’ to 6 = ‘none of the time’.^44^ Responses are summed to give scores for six sleep domains: SDS, somnolence (SOM), sleep adequacy (ADQ), snoring, awaking short of breath or with a headache, and two indices of sleep problems summarizing six (Index I) (SPI1) or nine (Index II) (SPI2) items.^45^ Multi-item scores show good internal consistency, with Cronbach’s alpha 0.71 to 0.81.^46^Supplementary Table 1 indicates which items contribute to each score, with some items contributing to more than one score. We define people to have the optimal sleep if 7 h ≤self-reported sleep duration ≤8.^47^

#### The Epworth Sleepiness Scale

The ESS^42^ assesses sleep propensity and daytime sleepiness.^44^ Participants rate how likely they are to doze off or fall asleep in eight common situations that vary in their soporific qualities, such as watching TV, talking to someone or lying down.^44^ Responses are on a 4-point scale ranging from 0 = ‘would never doze’ to 3 = ‘high chance of dozing’. Responses are summed to produce a total score ranging from 0 to 24, with higher scores indicating greater daytime sleepiness.^44^ The ESS has been shown to have good internal consistency (Cronbach *α* = 0.73–0.88) and test–retest reliability (correlation of measures across a 5-month interval = 0.82).^42^ A threshold of ≥11 as defined here (http://epworthsleepinessscale.com/about-the-ess/) is applied for determining ESS abnormal.

#### The Insomnia Severity Index

Insomnia severity is assessed with the ISI,^43^ a validated clinical measure that asks about symptoms of insomnia in the last 2 weeks. The first three items assess early, middle and late insomnia symptoms. The last four items measure sleep satisfaction/dissatisfaction, SDS noticeability, sleep worry and sleep interference with daily life, respectively. For these items, Likert scores of 0 represent ‘very satisfied’ or ‘not at all worried/noticeable/interfering’, whereas scores of 4 represent ‘very dissatisfied’ or ‘very much worried/noticeable/interfering’. Its internal consistency, concurrent validity and sensitivity to clinical improvements in insomnia patients are well established.^43^ Responses are summed to produce a total score ranging from 0 to 28, with higher scores indicating increasing insomnia severity. A threshold of ≥10 as defined here^48^ is applied for determining ISI abnormal.

All sleep scores are average scores across the multiple items and inverted (higher score means better sleep).

#### Sleep disorder diagnosis information

The presence of diagnosed sleep disorders, such as insomnia, restless leg syndrome and obstructive sleep apnoea, is determined by the questions ‘Have you ever been told by a doctor or other health professional that you have any of the following?’ and ‘Do you use a Continuous positive airway pressure (CPAP) machine or other appliance when you sleep to treat your sleep apnoea if you have apnoea?’.

### Apolipoprotein E, cognitive composites and cognitive status

Apolipoprotein E (*APOE*) genotype is expressed as a binary categorical variable, with participants classified as carriers (one or more ɛ4 alleles present) or non-carriers (no ɛ4 allele present).

As sleep may affect various aspects of cognition differently,^49^ we include five cognitive composite indices, reflecting the average of domain-specific standardized test scores (*Z*-scores) administered as part of the WRAP battery.^40^ The cognitive composites include working memory,^50^ immediate memory, delayed memory, executive function (EF)^51^ and a Preclinical Alzheimer Cognitive Composite (PACC).^52,53^ The tests contributing to each composite are shown in Supplementary Table 2.

Cognitive status is determined for each visit using a consensus review process that incorporated internal as well as published norms. A multi-disciplinary panel reviews cases to determine whether mild cognitive impairment or dementia was present.^54^

### Concurrent health at sleep assessment

#### Self-reported health measurements

Self-rated health^55^ is measured using a 5-point scale (1 = poor, 2 = fair, 3 = good, 4 = very good and 5 = excellent) in response to the question ‘How would you rate your current health?’. Depressive symptoms [20-item Center for Epidemiologic Studies Depression Scale (CES-D)]^56^ are completed by each participant. Self-rated memory is measured using a 7-point scale in response to the question ‘Overall, how would you rate your memory in terms of the kinds of problems that you have?’. The scores are summarized as 1–3 = major problems, 4 = neutral and 5–7 = no problems.

#### Objective health measurements

Two measures of obesity [BMI and waist–hip ratio (WHR)] are calculated. To evaluate BMI (/m^2^), height (m) and weight (kg) are measured. The WHR is calculated using the circumferences of the two target areas (waist and hip). The homeostatic model assessment of insulin resistance (HOMA-IR) is used to measure IR and is calculated as follows: fasting insulin (μU/mL) × fasting glucose (mmol/L)/22.5. A high HOMA-IR denotes low insulin sensitivity. HOMA-IR values are log-transformed into normally distributed values prior to analysis.

#### Medication use

Medication data is obtained at each visit through a combination of self-report, medical records and research staff review of medications brought to the study visit. The total number of prescriptions is counted for each participant at each visit, up to 15 per type.^57^

### Pittsburgh Compound B PET imaging

A subset of WRAP participants complete [^11^C]PiB^58^ amyloid PET imaging at the University of Wisconsin—Madison Waisman Brain Imaging Laboratory. Detailed imaging methods have been previously described.^59,60^ Amyloid burden is assessed as global cortical PiB distribution volume ratio (DVR)^20^ for continuous analyses, and one DVR threshold of ≥1.2 as defined previously^61^ is applied for determining PiB positivity (A+). Estimated amyloid chronicity (i.e. estimated years A+) is calculated at time of sleep assessment using previously published methods.^62,63^

### Statistical analysis

All analyses were performed in R version 4.0.0. For all analyses, significant omnibus tests (*P* < 0.05) were followed with unadjusted pairwise comparisons.

#### Aim 1 analyses

In preparation for our cluster analyses, all sleep variables were mean-centred and scaled such that higher scores indicated better sleep. We then used K-means cluster analysis to characterize subgroups of sleep variables in WRAP participants (*n* = 619), conducted using ‘factoextra’ package in R.^64^ The cluster assignment was based on the minimum distance (sum of the deviation of each variable) of a participant from the centroid of the cluster. The optimal number of clusters was identified using the elbow method by looking at the total within-cluster sum of square (WSS). To characterize the sleep group for each clustering-based subgroup of participants, the effect size ($\varepsilon^{2}$) of the sleep problems used in cluster analysis was noted in the right column of Supplementary Table 3. The relative contributions of the different problems in the grouping of participants were large, medium and small when $\varepsilon^{2}\;$≥ 0.26, $\varepsilon^{2}\;$≥ 0.08 and $\varepsilon^{2}\;$≥ 0.01, respectively.^65^

Given the high correlation among sleep variables, we conducted preliminary cluster analyses, sequentially excluding subsets of the scales and examining fit statistics and consistency across solutions. Based on the best WSS and Calinski–Harabasz Index values, the following subset of scales was selected in primary analyses: SPI1, SDS, ADQ, SOM, self-reported sleep duration, ESS and ISI.

To characterize how sleep groups differed across sleep characteristics, we used chi-square for categorical variables and Kruskal–Wallis tests for Likert-scale variables [median (Q1–Q3) reported]. *Post hoc* pairwise group differences at unadjusted *P* < 0.05 were reported.

Three sensitivity analyses were conducted to investigate the consistency of sleep group assignments and to examine whether between sleep group patterns in our outcomes were stable across different sample selection criteria. Alternative 1: we used LPA to characterize sleep subgroups (‘Mclust’ package in R). Briefly, LPA was a data-driven approach using continuous variables and indicators to identify subgroups of individuals. In this statistical approach, subgroup membership was determined by examining the pattern of interrelationships among indicator variables (maximizing homogeneity within each subgroup and heterogeneity between subgroups).^66^ Alternative 2 (cognitively unimpaired subset only): we reduced the original set to include only those who were cognitively unimpaired (*n* = 21 with mild cognitive impairment were removed; leaving *n* = 598), and K-means cluster analysis was used in this subset. Alternative 3 (expanded set with imputed ISI): as previously noted, the primary cluster analysis was based on the first visit with MOS, ESS and ISI. Since the MOS and ESS questionnaires were added to the battery several years before the ISI, we opted to enlarge ‘baseline sleep’ in sensitivity analyses to include those who had not yet completed an ISI but had completed MOS and ESS at least once. The imputation method used the sleep data on a person both before and after the ‘missing value’. The next observation carried backward assigned the person’s next known sleep score after the ‘missing’ one to the ‘missing value’. If the person did not have the next value, the last observation carried forward, assigned the person’s last previous known sleep score to the ‘missing value’, was used.^67^ The resulting enlarged set included *n* = 1237 available.

#### Aim 2 analyses

To analyse differences between sleep groups in demographic characteristics and concurrent health measures (*n* = 619), we used chi-square or Fisher’s exact test for categorical variables, analysis of variance (ANOVA) for continuous variables [mean (SD) reported] and Kruskal–Wallis tests for Likert-scale variables [median (Q1–Q3) reported]. *Post hoc* pairwise group differences at unadjusted *P* < 0.05 were reported. We excluded people who took insulin medication (*n* = 9) when comparing the HOMA-IR difference across the sleep groups (see Wallace *et al.*^68^). Linear regression was used to assess the relationship between sleep group and concurrent cognitive composite scores after adjusting for covariates [age, sex, education, WRAT3 reading score and the number of prior exposures to the cognitive tests (the practice effect)].

Similarly, to analyse differences between sleep groups and concurrent amyloid burden, we examined data from the subset that had completed at least one PiB PET study [*n*(%) = 108 (17.4%)]. Kruskal–Wallis tests were used to assess the difference between sleep groups in estimated concurrent global PiB DVR and amyloid chronicity, and Fisher’s exact test was used to analyse the concurrent amyloid PET status difference between sleep groups. In sensitivity analyses, we tested whether there was significant difference of amyloid burden at the most recent PET scan across the alternative sleep group assignments. In the imputed data set, 285 (23.0%) had at least one PiB PET scan, and we tested the difference in estimated concurrent and most recent global PiB DVR and amyloid chronicity among sleep groups.

We compared corrected Akaike information criteria (AICc) model fit statistics across otherwise identical models and considered |ΔAICc| values <2 to represent comparable models. Linear regression was performed for the association between sleep groups and concurrent cognitive composite scores after we removed stroke (*n* = 10), epilepsy/seizures (*n* = 13), multiple sclerosis (*n* = 5) and Parkinson’s disease (*n* = 2). Since *APOE* genotype associates with cognition, additional linear regression was performed including *APOE* e4 carriers in the model, and we compared model fits with the fits of the model in Aim 2 with the participants who have *APOE* data (*n* = 538). ΔAICc values were reported.

#### Aim 3 analyses

Last, we replicated analyses from an earlier WRAP PET publication.^20^ That study showed significant yet small associations between less adequate sleep, more sleep problems and greater SOM on the MOS and greater amyloid PET burden in Alzheimer’s disease–sensitive brain regions among 98 cognitively unimpaired adults (aged 62.4 ± 5.7 years) at their fourth WRAP visit. Participants were identified for the present analysis if they had completed WRAP Visit 4 (including sleep assessment), had completed a PiB PET scan and were non-demented; 315 individuals met these inclusion criteria. To match with the data set in Sprecher *et al*., we then excluded 95 people, leaving a sample size *n* = 220. We performed the same linear regression previously performed in Sprecher *et al*.,^20^ including age, sex, *APOE e4* genotype, family history of Alzheimer’s disease and BMI as covariates.

## Data availability

All data and materials used within this study will be made available, upon reasonable request, to research groups wishing to reproduce/confirm our results.

## Results

### Identifying sleep groups

A total of 619 participants were eligible for Aim 1 analyses. Mean (SD) sleep baseline age was 62.6 (6.7). Correlations among sleep variables are shown in Supplementary Fig. 1 and range from 0.87 (SPI1 and ADQ) to 0.15 (ESS and SDS).

#### Sleep groups identified by K-means cluster analysis (primary)

**Figure 1:**
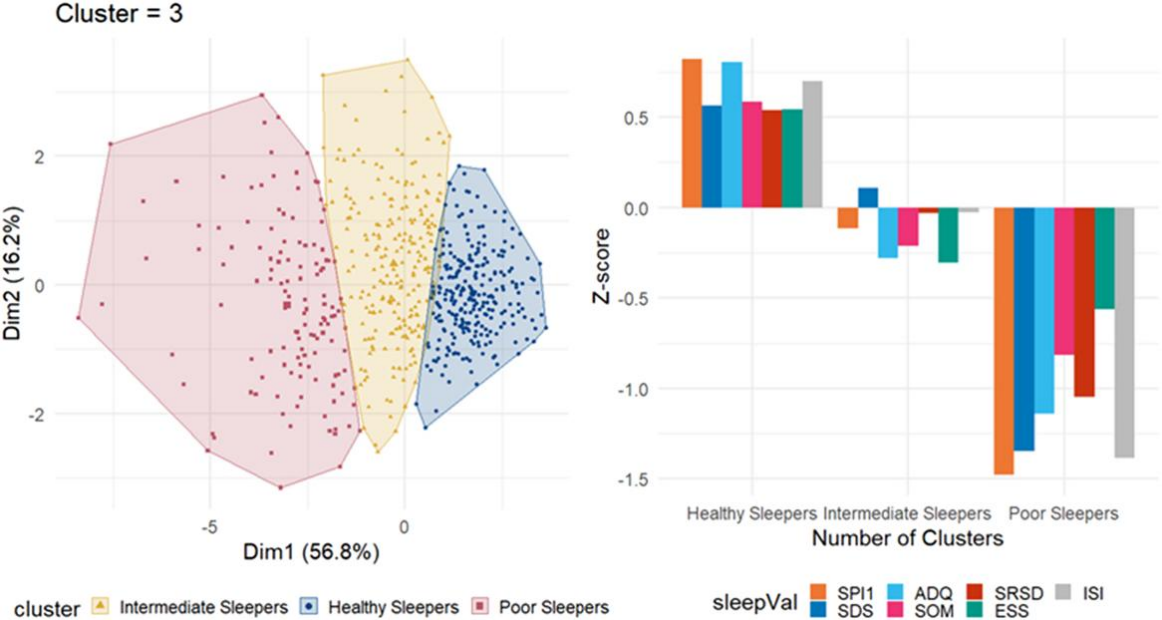
**K-means clustering of participants.** Left panel: Clusters are distributed along the principal components. Observations are represented by points in the plot. Right panel: Mean *Z*-scores of each variable within each group. The *Z*-scores are calculated for the current sample, yielding a sample sum of 0 and a standard deviation of 1; thus, the groups tend to approximately mirror each other around the *y* = 0 axis when the group sizes are similar. SPI1, Sleep Problem Index I; SDS, sleep disturbance scale; ADQ, sleep adequacy; SOM, somnolence; SRSD, self-reported sleep duration; ESS, Epworth Sleepiness Scale; ISI, Insomnia Severity Index. The optimal number of clusters given by the elbow method was 3. The total within-cluster sum of square (WSS) was 2282.454.

K-means cluster analysis identified three groups: healthy sleepers [HS, *n* = 262 (42.3%)], intermediate sleepers [IS, *n* = 229 (37.0%)] and poor sleepers [PS, *n* = 128 (20.7%)]. The cluster solution and differences in contributing variables were shown in Fig. 1. Differences across sleep groups were shown for all sleep variables in Supplementary Table 3. In *post hoc* pairwise comparisons, all sleep group pairs differed at *P* < 0.001. The relative contributions of all cluster analysis variables except ESS in the grouping of participants were large, and the three largest contributors were SPI1, ADQ and ISI. PS group also had a lower score on additional sleep variables, including more self-reported restless leg syndrome and apnoea.

As shown in Table 1, there were no significant differences between sleep groups on age, sex and *APOE* e4 carrier status. The PS group was slightly more racially diverse and had lower mean education years and lower WRAT3 reading.

**Table 1:** Demographic and baseline sleep concurrent health overall and by sleep group

	Healthy sleepers (*N* = 262)	Intermediate sleepers (*N* = 229)	Poor sleepers (*N* = 128)	*P*-value^a^	Difference pairs
Age (years) [mean (SD)]	63.24 (6.77)	62.25 (6.68)	62.07 (6.66)	0.151	
Male (%)	74 (28.2)	80 (34.9)	33 (25.8)	0.129	
Race (non-Hispanic White) (%)	25 (9.54)	191 (16.6)	93 (27.3)	<0.001	All pairs
College (%)	179 (68.3)	141 (61.6)	56 (43.8)	<0.001	PS versus HS, IS
*APOE* e4 carriers positive (%)	78 (33.2)	85 (41.5)	44 (44.9)	0.072	
WRAT3 reading [median (IQR)]	109.00 (102.00, 115.00)	109.00 (100.50, 115.00)	105.00 (98.00, 112.00)	<0.001	PS versus HS, IS
Concurrent health					
CES-D score [median (IQR)]	2.00 (1.00, 5.00)	6.00 (3.00, 11.00)	12.00 (7.00, 18.00)	<0.001	All pairs
SRH				<0.001	All pairs
Poor	0 (0.0)	4 (1.8)	2 (1.6)		
Fair	8 (3.1)	15 (6.6)	22 (17.3)		
Good	80 (30.7)	105 (46.5)	62 (48.8)		
Very good	131 (50.2)	88 (38.9)	35 (27.6)		
Excellent	42 (16.1)	14 (6.2)	6 (4.7)		
Num_prescription [median (IQR)]	2.00 (0.00, 4.00)	3.00 (1.00, 5.00)	4.00 (1.00, 7.00)	<0.001	All pairs
Self-rated memory				<0.001	All pairs
Major problems	11 (4.2)	33 (14.4)	31 (24.2)		
Neutral	35 (13.4)	44 (19.2)	30 (23.4)		
No problems	216 (82.4)	152 (66.4)	67 (52.3)		
Mild cognitive impairment (%)	5 (2.1)	8 (3.7)	8 (3.7)	0.089	

College = education years ≥16. CES-D, Center for Epidemiological Studies Depression Scale; SRH, self-rated health; Num_prescription, total number of prescriptions. ^a^Statistical tests: chi-square or Fisher’s exact test for categorical; analysis of variance (ANOVA) for continuous where mean (SD) reported; Kruskal–Wallis for continuous where median (Q1–Q3) reported and Likert-scale items. *Post hoc* pairwise group differences at unadjusted *P* < 0.05 were noted in the right-hand column. For example, HS versus IS and PS indicates group HS differed from group IS and group PS in separate pairwise comparisons.

#### Sleep groups identified in sensitivity analyses

As noted in the ‘Methods’ section, we used three additional approaches to identify alternative sleep group assignments. Each identified three groups with varying distributions. Alternative 1 (LPA): HS [*n* = 214 (34.6%)], IS [*n* = 198 (32.0%)] and PS [*n* = 207 (33.4%)] (Supplementary Fig. 2). Alternative 2 (restricting sample to the unimpaired subset): HS [*n* = 257 (43.0%)], IS [*n* = 220 (36.8%)] and PS [*n* = 121 (20.2%)]. Alternative 3 (larger imputed set): HS [*n* = 589 (47.6%)], IS [*n* = 429 (34.7%)] and PS [*n* = 219 (17.7%)]. The participant demographic, concurrent health and sleep variables in these 619 participants (original data), unimpaired subset and imputed set were similar (shown in Supplementary Table 4). The *Z*-scores of these sleep variables across the three groups identified by K-means and LPA in these data sets were similar (Supplementary Figs. 3 and 4). The disagreements between K-means and LPA cluster results and 598 participants in three data sets were shown in Supplementary Fig. 5.

### Examining associations between sleep groups and concurrent health

**Figure 2:**
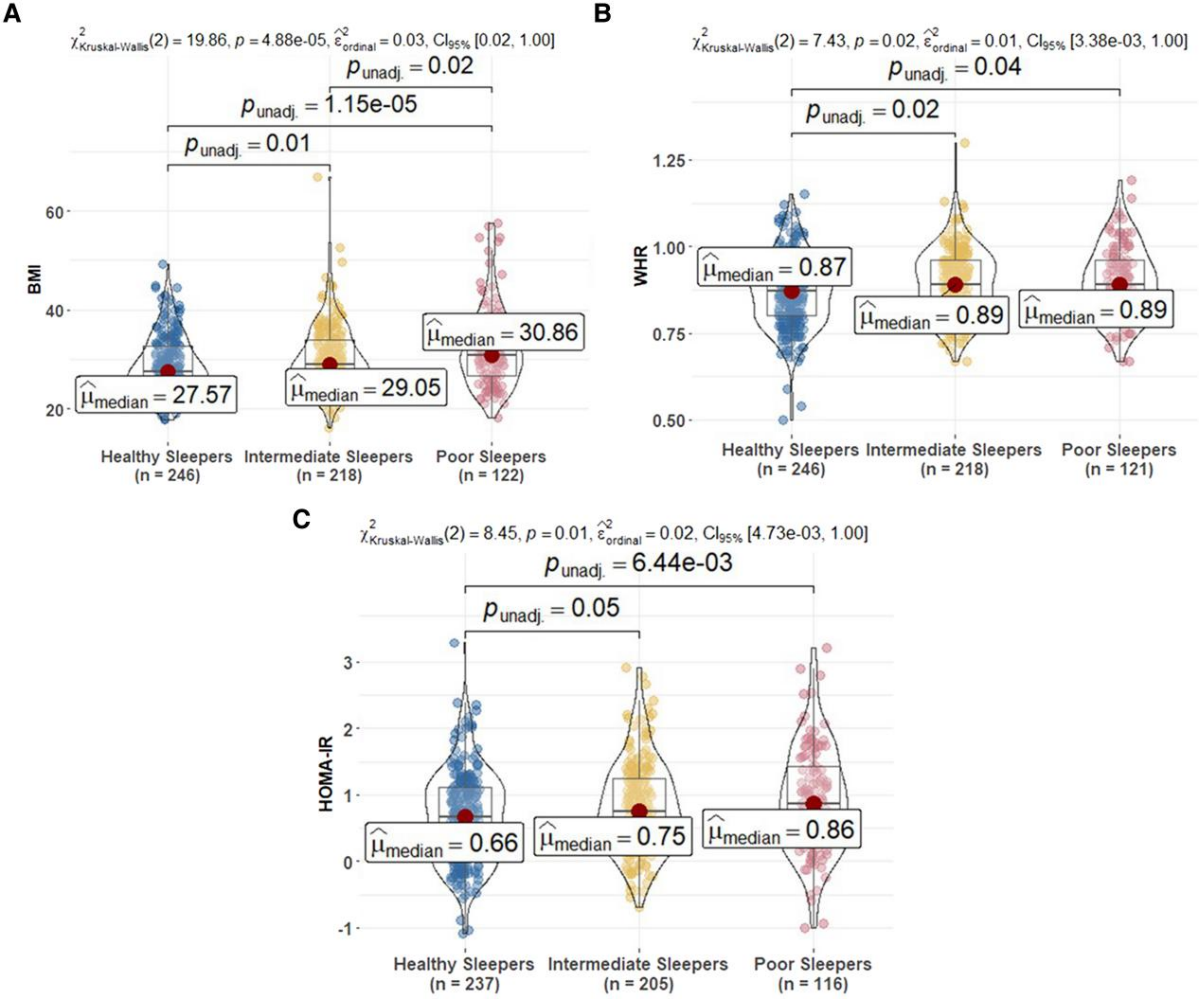
**Comparison of metabolic biomarkers at sleep baseline among sleep profiles.** (**A**) BMI, (**B**) WHR and (**C**) HOMA-IR. HOMA-IR is calculated by glucose (mg/dL) × insulin (mIU/mL)/405 (excluding people on insulin therapy). In the figure, HOMA-IR is log-transformed. BMI, body mass index; WHR, waist–hip ratio; HOMA-IR, homeostatic model assessment of insulin resistance. *Post hoc* significant pairwise group differences at unadjusted *P* < 0.05 are shown in the figure.

The PS group had the worse concurrent health, including self-reported depression, health and memory complaints, than the HS and/or IS groups (Table 1). PS had higher BMI, WHR and HOMA-IR than HS and IS (Fig. 2).

Sensitivity analyses comparing the concurrent health variables across sleep clusters described in the ‘Methods’ section showed that patterns for the LPA groups were the same in BMI compared with the primary K-means groups (Supplementary Fig. 6); patterns of association using the second and third clustering alternatives were consistent to those in the primary sleep group analyses (Supplementary Figs. 7 and 8); the only observed discrepancies were that differences between HS and PS for WHR (unimpaired subset) and between IS and PS for BMI (imputed ISI) were not significant.

### Examining associations between sleep groups and objective cognitive performance

**Figure 3:**
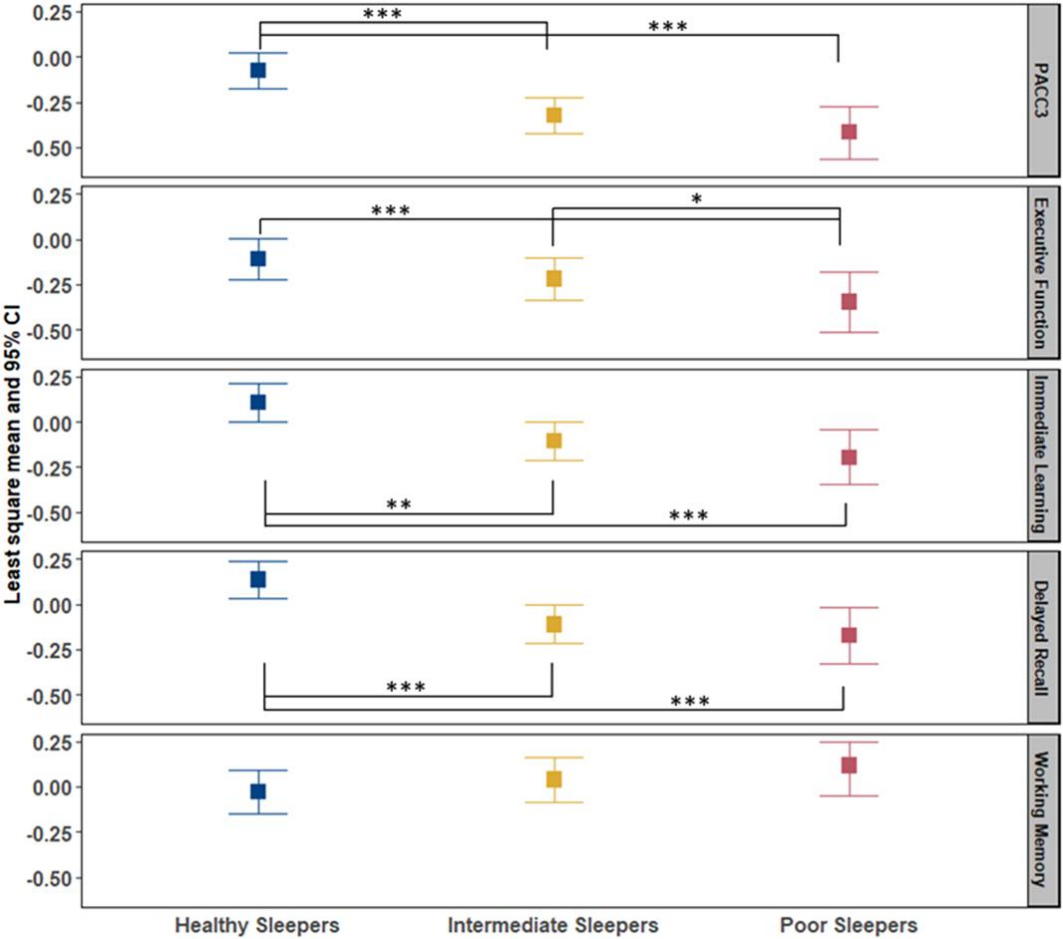
**Comparison of cognitive composite scores among sleep profile.** PACC3 = Preclinical Alzheimer’s Cognitive Composite (comprised of averaged *Z*-scores for three tests: Auditory Verbal Learning Test (AVLT) Total Learning, Logical Memory Delayed Recall and Digit Symbol Substitution); EF composite = averaged *Z*-scores for Stroop Color–Word, Trail Making Test Part B and Wechsler Adult Intelligence Scale—Revised (WAIS-R) Digit Symbol; immediate learning composite = averaged *Z*-scores for AVLT Total Learning, Wechsler Memory Scale-R Logical Memory-I and Brief Visuospatial Memory Test—Revised (BVMT-R); delayed recall composite = averaged *Z*-scores for AVLT Delayed Learning, Wechsler Memory Scale-R Logical Memory-II and BVMT-R Delayed. All composite scores are covariate-adjusted (age, sex, WRAT3 reading, college and practice effect). Analysis of covariance was used to compare the difference of composite scores in healthy sleepers (HS), intermediate sleepers (IS) and poor sleepers (PS). *P*-value *0.05, **0.01 and ***0.001.

All omnibus tests of sleep groups’ differences on cognitive composite scores were significant. After adjusting for covariates, PS performed worse on all cognitive outcomes except working memory (Fig. 3). The IS group performed worse on average than the HS group on PACC3 (Cohen’s *d* = 0.32), immediate learning (Cohen’s *d* = 0.27) and delayed recall (Cohen’s *d* = 0.31). The same pairwise differences were observed for PS versus HS with these effect sizes: PACC3, 0.51; immediate learning, 0.36; and delayed recall, 0.38. Last, the PS group performed worse on average than HS (effect size = 0.37) and IS (effect size = 0.25) on EF.

The number of participants with stroke, epilepsy/seizures, multiple sclerosis and Parkinson’s disease in HS/IS/PS group was 3/5/2, 3/5/5, 2/1/2 and 1/1/1, separately. After excluding these conditions, linear regression showed similar results in Aim 2 except the PS group versus IS group on EF was not significant. The effect size between IS and HS group increased from 0.32 to 0.36 on PACC3, from 0.27 to 0.30 on immediate learning and from 0.31 to 0.33 on delayed recall, and the effect size between PS and HS decreased by 0.06 on PACC3, 0.02 on immediate learning and 0.04 on delayed recall. In sensitivity analyses, linear regression was performed in alternative unimpaired subset and imputed data set. In the subset of primary data set (*n* = 538) who have *APOE* data, LM model of cognitive composite scores showed better fit except EF after adding the *APOE* e4 carriers to the model, including covariates and age terms (AICc decreased 1.2 on PACC3 model, AICc decreased 3.4 on immediate learning model, AICc decreased 3.7 on delayed recall model and AICc increased 0.15 on EF model). All the regression results were shown in Supplementary Tables 5–9.

### Examining associations between sleep groups and PiB PET amyloid

**Figure 4:**
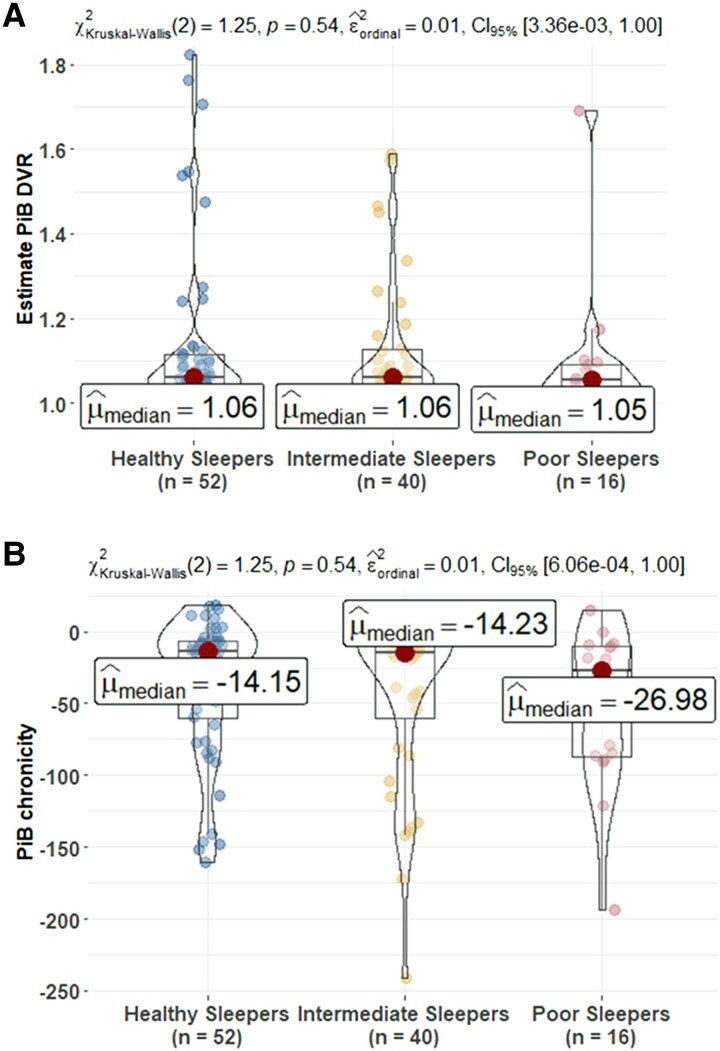
**Comparison of PET amyloid measures at sleep baseline among sleep profiles.** (**A**) Estimates build on the PiB DVR and (**B**) amyloid chronicity described in Koscik *et al*.^62^ The number (%) of PiB(+) in healthy sleepers (HS), intermediate sleepers (IS) and poor sleepers (PS) is 9 (17.3%), 7 (17.5%) and 1 (6.2) (Kruskal–Wallis *χ*^2^ = 1.26, *P* = 0.53). PiB(+) defined as any estimate PiB DVR within a person ≥1.2. The mean (95% CI) estimate age PiB positive in HS, IS and PS is 77.61 (73.09, 81.97), 78.28 (75.51, 81.44) and 80.93 (75.61, 85.38). PiB, Pittsburgh Compound B; DVR, distribution volume ratio. *Post hoc* significant pairwise group differences at unadjusted *P* < 0.05 are shown in the figure.

In the data set used for the primary analyses (*n* = 619), 109 (17.4%) had at least one PiB PET scan. There were no significant differences between sleep groups on estimate DVR at the time of sleep assessment (Fig. 4). The difference in amyloid status between sleep groups was not statistically significant. After replicating the analyses using the sleep clusters from LPA (Supplementary Fig. 9), in the unimpaired subset (Supplementary Fig. 10) or in the larger PiB PET scan sample (*n* = 287) (Supplementary Fig. 11), the results were consistent.

### Replication study comparing current data set with previous results

Participant demographic and cognitive characteristics in the prior^20^ study and in this replication study with bigger sample size were compared in Table 2. The mean age in this replication study was 63.7 years (SD = 5.78, range = 50.2–74) at the time of the PiB PET scan, 1.3 years on average older than previous study. Mean interval between PET scan and questionnaire completion was 1.28 (SD 1.10) years, 0.59 years longer than previous study, and the association did not change when interval was added as a covariate. Sex, years of education, BMI, CES-D and cognitive scores were similar.

**Table 2:** Participant characteristics in original and expanded PET PiB samples

	Sprecher *et al*.^a^ (*N* = 98)	Replication sample (*N* = 220^b^)
Age at PiB PET scan, years	62.4 (5.7; 50–73)	63.7 (5.8; 50.2–74.0)
Age at sleep assessment, years	63.0 (5.6; 51–73)	63.6 (5.6; 51.4–73.7)
Interval between PiB PET scan and sleep assessment, years	0.69 (0.98; 0–3.7)	1.28 (1.10; 0–3.6)
Female, %	67.3	66.0
*APOE e4* positive, %	34.7	38.6
FH positive, %	75.5	74.1
BMI, kg/m^2^, mean (SD)	28.7 (5.7)	28.6 (5.7)
Education, years	16.6 (2.8; 12–25)	16.2 (2.1; 12–20)
CES-D	5.78 (5.48; 0–27)	5.76 (5.56; 0–27)
MMSE	29.31 (1.22; 23–30)	29.38 (0.96; 24–30)
AVLT total	50.21 (8.66; 30–67)	50.51 (8.68; 28–67)
AVLT delayed recall	10.36 (2.96; 0–15)	10.63 (2.83; 1–15)
Trails A time^c^	10.11 (2.18; 5–17)	10.44 (2.45; 5–17)
Trails B time^c^	10.26 (2.51; 6–17)	10.56 (2.48; 3–17)
Digit symbol scaled score^c^	13.35 (2.1; 9–19)	13.30 (2.1; 8–19)
PiB DVR		1.14 (0.18; 0.9–2.07)

All values are mean (SD; range) except where indicated. *APOE e4*, the epsilon 4 allele of the apolipoprotein E gene; FH, family history of Alzheimer’s disease; BMI, body mass index, CES-D, Center for Epidemiological Studies Depression Scale; MMSE, Mini-Mental State Exam; AVLT, Auditory Verbal Learning Test. ^a^The details are in Sprecher *et al*. paper.^20^^b^Replication sample (exclusion: the interval between age at PiB PET scan and sleep assessment greater than 3.7 years (*n* = 75), older age at PiB PET scan and sleep assessment (i.e. age >74, *n* = 10), higher CES-D score (i.e. >27, *n* = 6) and higher Auditory Verbal Learning Test (AVLT) total score (i.e. >67, *n* = 4). ^c^Scaled for age and sex.

**Figure 5:**
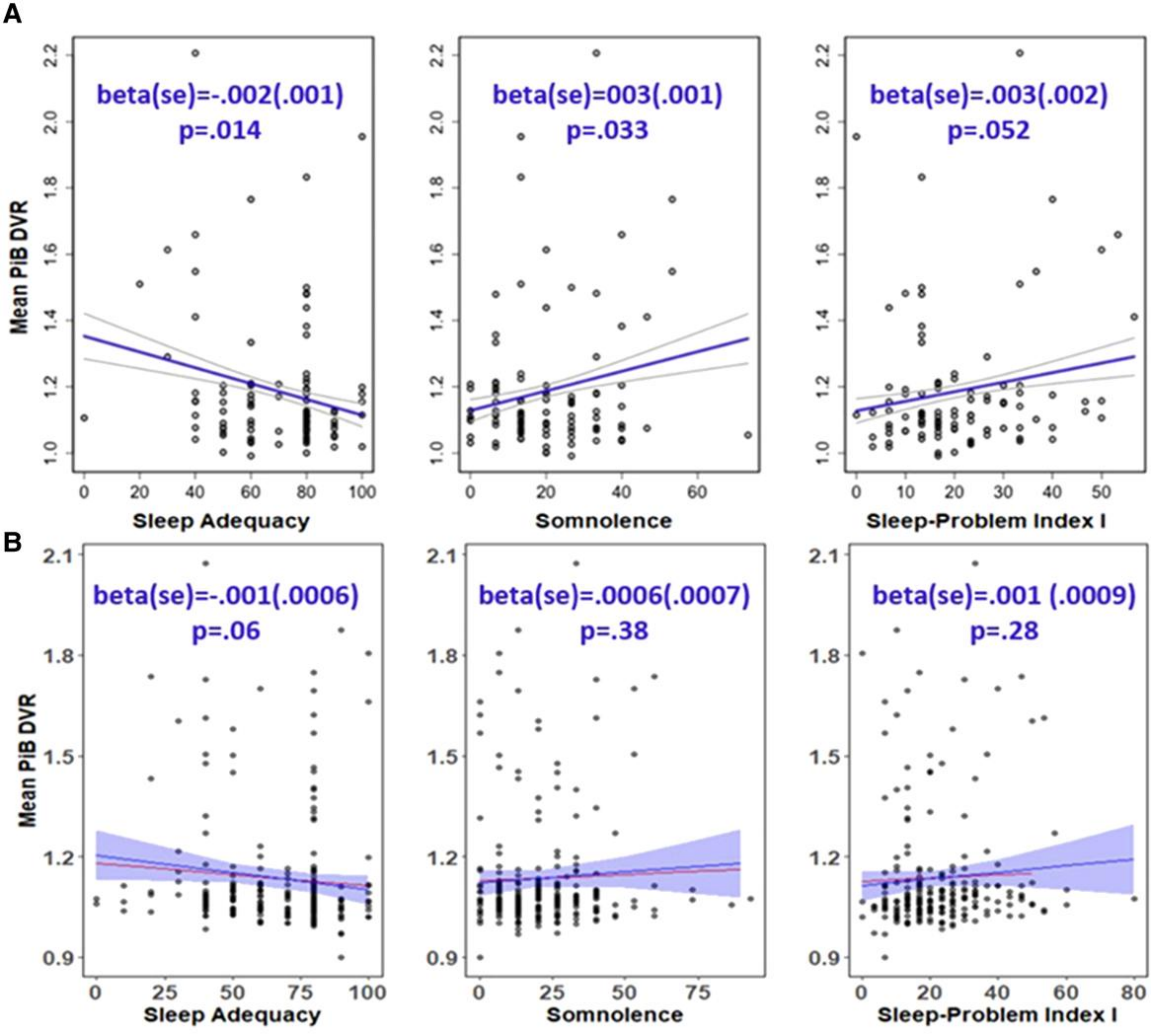
**Association between sleep adequacy, somnolence, Problem Index I scores and mean PiB DVR.** (**A**) Results from Sprecher’s paper (*top* panel). (**B**) Replication results (*bottom* panel). Raw data is plotted, and the regression line with ribbon is adjusted for age, sex, the epsilon 4 allele of the apolipoprotein E gene, family history of Alzheimer’s disease and body mass index (the line without ribbon is the regression line without covariates adjustment). Per prior research,^20^ the sleep scores (*x*-axis) were scaled to a range of 0–100 with higher values indicating more of the concept being measured (higher score is better for sleep adequacy, worse for somnolence and Problem Index I scores). The mean PiB DVR value (*y*-axis) is distribution volume ratio, which reflects the equilibrium distribution of PiB. The test statistic values alongside *P*-values from separate regression models with covariate adjustment are shown in the figure. Results were considered statistically significant when *P* < 0.05. DVR, distribution volume ratio; PiB, Pittsburgh Compound B.

After adjusting for covariates (age, sex, *APOE e*4 status, family history of Alzheimer’s disease and BMI), poorer sleep was not significantly associated (*P* > 0.05) with greater PiB DVR on any of the sleep measures examined. The results were summarized in Supplementary Table 10 together with the Sprecher results. Scale score versus PiB DVR were plotted in Fig. 5 for the three variables with smallest *P*-values in the Sprecher *et al*. paper. While the previous publication reported significant associations between PiB DVR and ADQ [beta (SE) = −0.002 (0.001), *P* = 0.014] and SOM [beta (SE) = 0.003 (0.001), *P* = 0.033], these associations were weaker and NS in the current PiB PET data set [ADQ beta (SE) = −0.001 (0.0006), *P* = 0.06; SOM beta (SE) = 0.0006 (0.0007), *P* = 0.38)].

## Discussion

In this study, we applied person-centred data-driven analysis techniques to cognitively unimpaired WRAP participants’ self-reported sleep characteristics. This novel approach allowed us to identify more homogeneous subgroups that differed on self-reported sleep quality, insomnia and daytime sleepiness. We then demonstrated how these HS, IS and PS subgroups also differed in concurrent health, cognition and amyloid PET. Four major findings resulted, and each finding was discussed in greater detail below. Overall, the evidence suggested that conceptualizing sleep as a single dimension or the total score of multiple items could not fully capture the changes and variability in self-reported sleep patterns and supported the multi-dimensional sleep health perspective.^69^ Participants in the HS group reported better concurrent mental and physical health and demonstrated better cognition than those in the other groups. The PS group was more likely to report increased depressive symptoms, more physical health problems and worse cognition. However, there were no significant associations between the sleep variables and amyloid PET measures.

### Finding 1: sleep groups differed on self-reported sleep quality and select demographics

Three groups of participants were identified which were heterogeneous with respect to self-reported sleep characteristics. The group with the highest proportion of participants was HS (42.3%), which exhibited sufficient standings on all measured sleep characteristics. MOS scales SPI1, ADQ and ISI were the three sleep dimensions that contributed most to the cluster analysis. The PS group with more sleep problems, lower ADQ and more serious insomnia was slightly more racially diverse and had overall lower education levels and lower estimated premorbid ability level. A review of the association between race/ethnicity and sleep patterns summarized that the racialized group had objectively measured and self-reported worse sleep duration and quality than Whites.^70^ Other studies also reported race and education associations with sleep quality.^34,71-73^ For example, Stamatakis and colleagues^71^ found that short sleep duration was more common among those with lower education levels and among racialized racial and ethnic groups. Racialized groups were more likely to experience social disadvantage due to historical and contemporary forms of race-based institutional and interpersonal discriminatory policies and practices.^70^ The social disparities in sleep patterns provided further evidence that these disparities might be associated with disparities in other areas, such as cardiovascular and metabolic health.^74,75^ But more data were needed to assess the social disparities in sleep. There were no significant differences between sleep groups in age, sex and *APOE* e4 carriers. One study^32^ reported that younger men were more likely than their older counterparts to be assigned to the ‘poor sleep quality’ class. The mean age in that study was 42.7, which was much younger than our study. A review of recent studies showed women tend to experience the most significant sleep problems during the peri-menopausal period.^76^ However, given the age of our sample, nearly all women would have been post-menopausal at the time of the sleep data. Our study was consistent with Drogos *et al*.^77^ who reported no relationship between the presence of *APOE* e4 allele and subjective sleep complaints in a healthy population screened for dementia.

### Finding 2: sleep groups differed on concurrent health outcomes and subjective reports of functioning

The PS group had higher measured BMI, WHR and HOMA-IR than the HS and IS groups. These associations were also reported in other studies using variable-centred approaches.^78-80^ For example, shorter sleep duration was associated with higher BMI in a sample of 1042 individuals from Brazil, including both genders (20–80 years)^80^; partial sleep deprivation during only a single night induced IR in multiple metabolic pathways in nine healthy subjects.^79^ We also observed associations between sleep groups and concurrent self-reported health, depression and memory, such that those with the poorest self-reported sleep characteristics were worse on all these measures. Again, these results were consistent with other studies that used variable-centred approaches (e.g. Tsapanou *et al.*, Paunio *et al.* and Carpi et al.^23,81,82^). For example, the onset of poor sleep predicted incident depression in a sample of 12 063 individuals using logistic regression models.^81^ But our study showed the co-aggregation of these risk variables using person-centred approaches. Some associations were found using different person-centred approaches in older adults.^19,35^ For example, Yu and colleagues distinguished four sleep profiles based on self-report measures of sleep problems: inadequate sleep, disturbed sleep, trouble falling asleep and multiple problems, using latent class analysis in a community sample of elderly (mean age 67 years). The ‘multiple problems’ group had significantly higher levels of depression and anxiety relative to the control group. However, our study was in a longitudinal preclinical study enriched with Alzheimer’s disease parental history from midlife, which was important to show these associations in this sample.

### Finding 3: sleep groups differed in objective cognitive performance

After adjusting for covariates (age, sex, college and WRAT3 reading score), PS performed worse on all cognitive outcomes except working memory. Poor sleep was considered a potential risk factor for cognitive decline,^83,84^ extreme sleep durations in later life were associated with worse average cognition in female nurses aged 70 and older free of stroke and depression at the initial cognitive assessment and poor sleep quality was associated with mild cognitive impairment in a group of 1793 participants (51% men; 63.8 ± 7.5 years) of the population-based Heinz Nixdorf Recall study. Observational studies^3,5,85-87^ with self-report sleep measures supported links between sleep and cognitive decline using variable-centred approaches. But the results were mixed. For example, one study found self-reported poor sleep quality was related to cognitive changes, whereas daytime sleepiness was not related.^18^ In contrast to other studies,^14,15,88^ McSorley *et al*.^9^ did not find evidence that self-reported sleep duration was a significant contributor to cognitive function. Cross *et al.*^89^ found adults with probable insomnia disorder exhibited declarative memory deficits compared with insomnia symptoms only or no insomnia symptoms; however, adults with insomnia symptoms exhibited better performance on a task of mental flexibility than both other groups. However, these studies assumed that all individuals at certain levels of risk factors were at equal risk of adverse outcome and therefore the association between a risk factor and outcome was the same across the entire population.

### Finding 4: sleep groups were not associated with PET measures of amyloid

No significant associations between sleep and amyloid PET measures were found using our person-centred approach or when we repeated a previous variable-centred analysis. Specifically, a previous study from this cohort found that in cognitively healthy adults (*n* = 98), less adequate sleep, more sleep problems and greater SOM were associated with higher amyloid burden in the angular gyrus, frontal medial orbital cortex, cingulate gyrus and precuneus when these individual sleep variables were included in separate models that adjusted for age, sex, *APOE e4*, family history of Alzheimer’s disease and BMI.^20^ In Aim 3, using the larger sample size now available, none of the individual sleep variables were significantly associated with amyloid burden. As shown in Fig. 5, we had a wider range of values on the SOM and the SPI1 than the values in the sample for the 2015 paper; associations remained non-significant after removing these higher points in sensitivity analyses. Similar to our current results, a study of 143 community-dwelling participants aged ≥70 years^12^ found no significant relationship between amyloid-PET burden and nighttime sleep duration, daytime sleep duration, 24-h sleep duration, naps, restless leg syndrome, daytime sleepiness, insomnia symptoms or sleep apnoea risk before and after adjustment for *APOE e4* and depressive symptoms using logistic regression models. Conversely, a cross-sectional study of 184 cognitively normal participants older than 60 years found that longer sleep latency was associated with PET-measured higher amyloid burden, independent of the *APOEe*4 status.^90^ Another cross-sectional study conducted by Spira *et al*.^91^ studied 70 community-dwelling subjects (mean age 76) and found a greater amyloid-β burden associated with both self-reported shorter sleep duration and poorer sleep quality using regression models. All these studies included older people than ours and used variable-centred methods with different sleep variables. Another study^92^ showed that greater amyloid-β burden was linked to significantly greater self-reported sleep problems and/or a significantly greater mismatch between participants’ subjective evaluation of sleep, relative to their actual objective sleep. As a result, individuals expressed lower subjective (perceived) sleep quality than their objective quality of sleep showed. Given the inconsistent associations between sleep characteristics and amyloid, more studies with both subjective and objective sleep measurements are needed to understand whether amyloid development is associated with specific sleep problems (such as Obstructive sleep apnea) or profiles of problems.

The current findings show the heterogeneity in self-reported sleep characteristics and support the importance of establishing good sleep among late middle-aged non-demented adults. Sleep could be a risk factor for mental and physical health, and objective cognition, but not amyloid burden, which has implications in clinical trial design and early intervention or prevention efforts. Subjective sleep measures could be a mediator or moderator in the health- and cognition-related research. In addition, clustering techniques should be considered when looking at the association between a risk factor and outcomes.

### Strengths

The strength of this study was the use of a novel analytic approach to leverage the underlying heterogeneity in self-reported sleep characteristics, identifying distinct groups of sleep subtypes among late middle-aged adults in a large prospective study. Moreover, the present study examined the association between SDS and five different cognitive domains. In addition, we used several strategies to investigate the robustness of our results. For example, we applied two person-centred methods to define the different sleep profiles to different cross-sectional subsets and observed that results were consistent with our primary approach.

### Limitations

First, this cross-sectional study does not determine whether poor sleep profiles precede cognitive decline (causation cannot be inferred). Considering the bi-directional relationship between sleep and cognitive decline,^93^ more severity of behavioural problems and cognitive impairment might actively worsen sleep, and poorer sleep might in turn worsen cognitive and physical functions. Second, similar to most of the previous studies,^13,14,91,94-96^ this study was based on self-reported information rather than objective measurement, which can be affected by reporting bias. For example, self-reported sleep time tended to overestimate sleep time.^97^ In some cases, self-reported sleep measures were only modestly correlated or even uncorrelated with objective sleep measures.^98^ Since objective and subjective measures of sleep have been shown to correlate differently with Alzheimer’s disease biomarkers,^92^ both are important to study in understanding Alzheimer’s disease–related decline. In the future, we hope to add objective sleep measurements to our study. However, we included the broadest array of health, mood, cognition, and other variables. Finally, the imputation method in sensitivity analysis was potentially flawed if the missingness is not random. Other imputation methods like multiple imputation could be used in the future. While our approach was useful in consolidating sleep measures into groups with common patterns, it is possible that some sleep characteristics have synergistic associations with cognition that are not captured by our approach. A key next step is to determine if these sleep trajectories align with more objective indicators of individuals’ sleep patterns and understand how an intervention impacts the meaning of the results. Future studies with larger sample size on sleep characteristics and cognition, also the pathologies of Alzheimer’s disease, not only amyloid plaque, but neurofibrillary tangles, are needed.

## Conclusion

This study indicates that clustering techniques can be used to identify sleep characteristic subtypes which are associated with concurrent mental, physical and cognitive health, but not beta amyloid. Although not all PS will develop mild cognitive impairment or progress to clinical dementia, this group appears to be at increased risk of cognitive decline. Future research will follow this group over time and will also examine how other risk factors differ between sleep groups. The ability to identify persons’ risk factors has implications for clinical trial design and early intervention or prevention efforts.

## Supplementary Material

fcad039_Supplementary_Data

## Abbreviations

ADQ =: sleep adequacy
AICc =: corrected Akaike information criteria
*APOE* =: apolipoprotein E
BMI =: body mass index
CES-D =: Center for Epidemiologic Studies Depression Scale
DVR =: distribution volume ratio
EF =: executive function
ESS =: Epworth Sleepiness Scale
HOMA-IR =: homeostatic model assessment of insulin resistance
HS =: healthy sleepers
IS =: intermediate sleepers
ISI =: Insomnia Severity Index
LPA =: latent profile analysis
MOS =: Medical Outcomes Study Sleep Scale
PACC =: Preclinical Alzheimer’s Cognitive Composite
PiB =: Pittsburgh Compound B
PS =: poor sleepers
SDS =: sleep disturbance scale
SOM =: somnolence
SPI1,2 =: Sleep Problems Index 1,2
WHR =: waist–hip ratio
WRAP =: Wisconsin Registry for Alzheimer’s Prevention
WSS =: within-cluster sum of square
