# Supplementary material for: Associations between self-reported sleep patterns and health, cognition and amyloid measures: results from the Wisconsin Registry for Alzheimer’s Prevention
Source: Brain Commun. 2023 Feb 24;5(2):fcad039. doi: 10.1093/braincomms/fcad039 (PMC9999364; doi:10.1093/braincomms/fcad039)
Supplement: fcad039_Supplementary_Data [file fcad039_supplementary_data.pdf]

Supplementary Table 1. Mapping of questions (rows) to MOS sleep scales (columns)

Raw scores	Sleep Problems Index I	Sleep Problems Index II	Sleep Disturbance	Snoring	Waking short of breath	Sleep Adequacy	Somnolence	Sleep Hours
**During the past 4 weeks…**								
1. How long did it usually take for you to fall asleep? [a]		X	X					
2. On the average, how many hours did you sleep each night? [b]								X
**How often did you…**[c]								
3. feel that your sleep was not quiet (moving restlessly, feeling tense, speaking, etc., while sleeping)?		X	X					
4. get enough sleep to feel rested upon waking in the morning?	RX	RX				RX		
5. awaken short of breath or with a headache?	X	X			X			
6. feel drowsy or sleepy during the day?		X						
7. have trouble falling asleep?	X	X	X					
8. awaken during your sleep time and have trouble falling asleep again?	X	X	X					
9. have trouble staying awake during the day?	X	X					X	
10. snore during your sleep?				X				
11. take naps (5 minutes or longer) during the day?							X	
12. get the amount of sleep you needed?	RX	RX				RX		

[a]Possible responses were 15 minute increments from 1 = "0–15' to 5 = "More than 60". [b]Responses were free-entry. [c]Responses were on a 6-point scale ranging from 1="All" to 6="None". [a,b,c,d]Responses were rescaled. NOTE. X in a cell indicates that the questions represented in that row contributed to that column's scales. RX indicates that item included in scale

Supplementary Table 2. Mapping of tests (rows) to five cognitive composites (columns)

Raw scores	Working memory	Immediate learning	Delayed recall	Executive function	PACC3
Rey AVLT Total	-	X	-	-	X
Rey AVLT Delayed	-	-	X	-	-
WMS-R Logical Memory-I	-	X	-	-	-
WMS-R Logical Memory-II	-	-	X	-	X
BVMT-R Total	-	X	-	-	-
BVMT-R Delayed	-	-	X	-	-
Stroop Color-Word	-	-	-	X	-
TMT Part A	-	-	-	-	-
TMT Part B	-	-	-	X	-
WAIS-R Digit Symbol	-	-	-	X	X
Digit span Forward	X	-	-	-	-
Digit span Backward	X	-	-	-	-
Letter-Number Sequence	X	-	-	-	-

Abbreviations: PACC, preclinical Alzheimer's cognitive composite; AVLT (Schmidt 1996), Auditory-Verbal Learning Test; BVMT-R (Benedict 1997), Brief Visuospatial Memory Test-Revised; Stroop Color-Word (Trenerry et al. 1989), Stroop test, Color-Word Interference; TMT (Reitan 1958), Trail Making Test; WMS-R (Wechsler 1987), Wechsler Memory Scale-Revised; WAIS-R Digit Symbol (Wechsler 1997), Digit Symbol subtest of the Wechsler Adult Intelligence Scale-Revised.
NOTE. X in a cell indicates that the test represented in that row contributed to that column's composite.

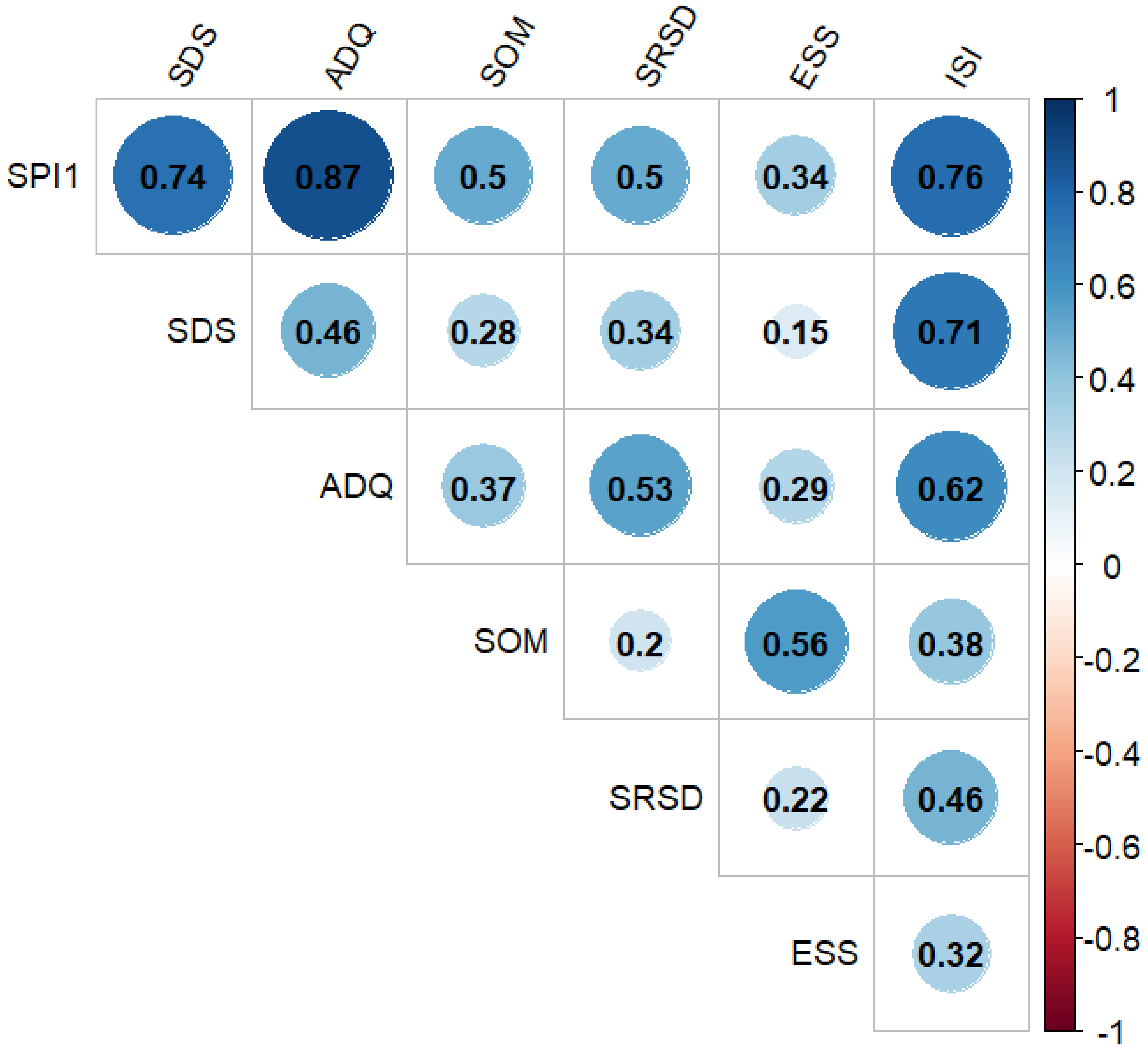

Supplementary Figure 1. Spearman correlations between sleep variables in cluster analysis. Numbers represent Spearman's rho. T test is used to test whether the correlation is due to chance or not, all pairs of correlation are signifiant ($p < 0.001$). Abbreviations: SPI1, Sleep-Problem Index I; SDS, Sleep Disturbance scale; ADQ, Sleep adequacy; SOM, Somnolence Scale; SRSD, Self-reported sleep duration; ESS, Epworth Sleepiness Scale; ISI: Insomnia Severity Index.

Supplementary Table 3 Differences in sleep problems between sleep profiles based on cluster analysis

	Healthy Sleepers (N=262)	Intermediate Sleepers (N=229)	Poor Sleepers (N=128)	Effect size(ϵ^2) [#] /P value[a]	Post hoc
**Cluster Analysis Variables**					
**SPI1 (median [IQR])**	5.33 [5.17, 5.50]	4.67 [4.50, 4.83]	3.67 [3.50, 4.00]	0.760/<0.001	
**SDS (median [IQR])**	5.00 [4.75, 5.50]	4.75 [4.25, 5.25]	3.50 [2.75, 4.00]	0.447/<0.001	
**ADQ (median [IQR])**	5.00 [5.00, 5.38]	3.50 [3.50, 4.50]	3.00 [2.00, 3.00]	0.609/<0.001	
**SOM (median [IQR])**	5.33 [5.00, 5.67]	4.67 [4.33, 5.33]	4.33 [3.33, 5.00]	0.311/<0.001	All comparisons
**SRSR (median [IQR])**	8.00 [7.00, 8.00]	7.00 [6.00, 8.00]	6.00 [5.00, 6.00]	0.372/<0.001	
**ESS (median [IQR])**	2.50 [2.38, 2.75]	2.12 [1.75, 2.38]	2.00 [1.59, 2.38]	0.217/<0.001	
**ISI (median [IQR])**	4.71 [4.43, 4.86]	4.14 [3.71, 4.43]	3.29 [2.86, 3.61]	0.585/<0.001	
**Additional Sleep Variables**				**P value** [a]	
**SPI2 (median [IQR])**	5.22 [5.00, 5.44]	4.56 [4.33, 4.89]	3.67 [3.33, 3.89]	<0.001	All comparisons
**SNR (median [IQR])**	5.00 [4.00, 6.00]	4.00 [3.00, 5.00]	4.00 [3.00, 5.00]	<0.001	HS vs IS, PS
**SOB (median [IQR])**	6.00 [6.00, 6.00]	6.00 [5.00, 6.00]	6.00 [5.00, 6.00]	<0.001	All comparisons
**Insomnia (%)**				<0.001	All comparisons
**No**	258 (98.9)	217 (95.2)	96 (75.0)		
**Yes**	3 (1.1)	9 (3.9)	27 (21.1)		
**Don't Know**	0 (0.0)	2 (0.9)	5 (3.9)		
**Restless leg syndrome(%)**				0.005	HS vs IS, PS
**No**	249 (95.4)	201 (87.8)	108 (84.4)		
**Yes**	10 (3.8)	24 (10.5)	18 (14.1)		
**Don't Know**	2 (0.8)	4 (1.7)	2 (1.6)		
**Apnea status (%)**				0.011	HS vs IS, PS
**No apnea**	226 (88.3)	180 (81.1)	89 (73.6)		
**Apnea with device**	22 (8.6)	32 (14.4)	24 (19.8)		
**Apnea no device**	8 (3.1)	10 (4.5)	8 (6.6)		
**ESS normal[b] = Yes (%)**	259 (98.9)	188 (82.1)	89 (69.5)	<0.001	All comparisons
**ISI normal[c] = Yes (%)**	182 (69.5)	54 (23.6)	0 (0.0)	<0.001	All comparisons
**Optimal Sleep[d] = Yes (%)**	219 (83.6)	137 (59.8)	19 (14.8)	<0.001	All comparisons

[*]All scores are average scores across the multiple items, and inverted (Higher score means better sleep).

[+]Medical Outcomes Study Sleep Scale (MOS): SPI1, Sleep-Problem Index I, 6 items; SPI2, Sleep-Problem Index II, 9 items; SDS, Sleep Disturbance scale, 4 items; SNR, Snoring Scale, 1 item; SOB, Sleep short of breath or headache, 1 item; ADQ, Sleep adequacy, 2 items; SOM, Somnolence Scale, 3 items; SRSD, Self-reported sleep duration, 1 item.
Epworth Sleepiness Scale (ESS): 8 items. Insomnia Severity Index (ISI): 7 items. *Spritzer, K. L. & Hays, R. D. (2003, November). MOS Sleep Scale: A Manual for Use and Scoring, Version 1.0. Los Angeles, CA.*

[#]To characterize the sleep profile for each clustering-based subgroup of participants, Effect size (ϵ^2) of the sleep problems used in cluster analysis is noted in the right column. The relative contributions of the different problems in the grouping of participants are large, medium and small when $\epsilon^2 \geq 0.26$, $\epsilon^2 \geq 0.08$, $\epsilon^2 \geq 0.01$.
[a]Statistical tests: chi-square or Fisher's exact for categorical; Kruskal-Wallis for continuous and Likert-scale items.
[b]Cut-off ESS = 11 (recoded and scaled ESS value is 1.75), http://epworthsleepinessscale.com/about-the-ess/
[c]Cut-off ISI = 10 (recoded and scaled ISI value is 4.57), *Morin, Charles M., et al. Sleep 2011: 601-608.*

[d]Optimal Sleep = Yes if 7 hours <= Self-reported sleep duration <=8, *Wolfe, F., Michaud, K. and Li, T., 2006. The Journal of Rheumatology, 33(10), pp.1942-1951.*

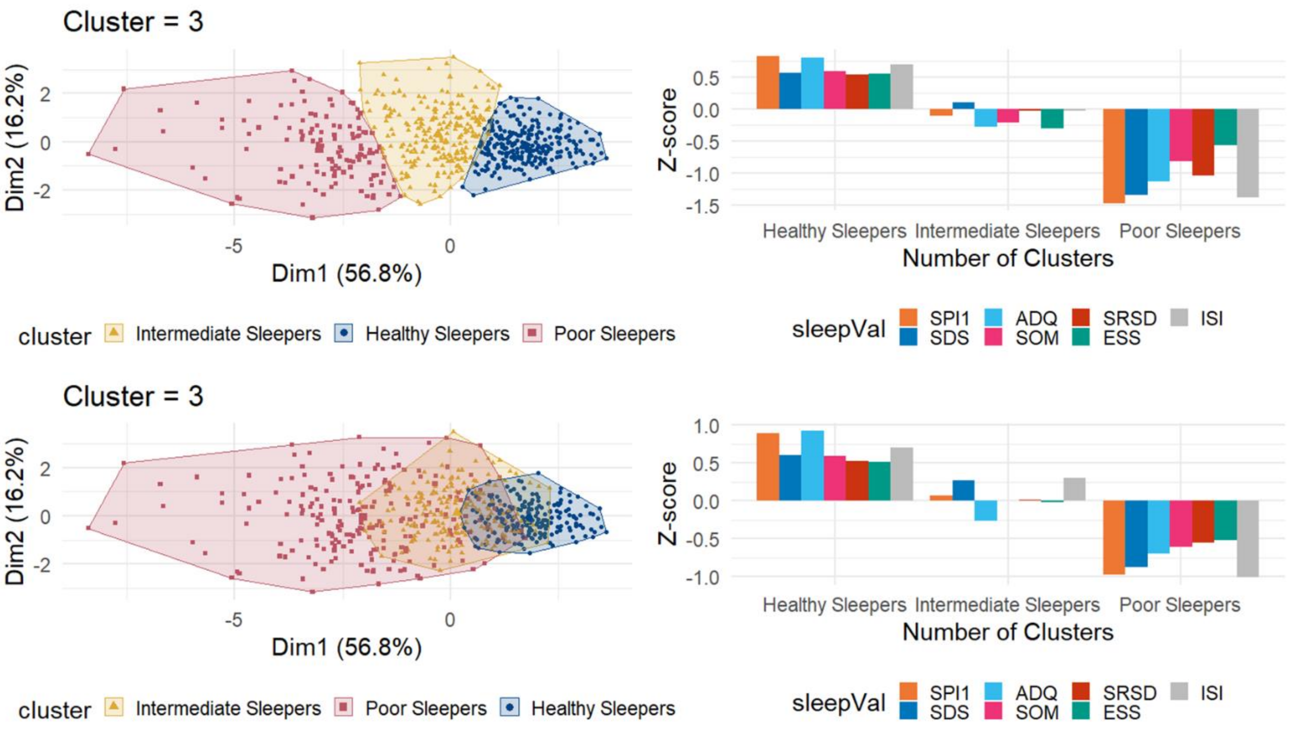

Supplementary Figure 2. Kmeans and Latent profile analysis clustering of participants (n=619). Left panel: Clusters are distributed along the principal components. Observations are represented by points in the figure. Right panel: Mean z-scores of each variable within each group. The z-scores are calculated for the current sample, yielding a sample sum of 0 and a standard deviation of 1, thus the groups tend to approximately mirror each other around the y = 0 axis when the group sizes are similar. Abbreviations: SPI1, Sleep-Problem Index I; SPI2, Sleep-Problem Index II; SDS, Sleep Disturbance scale; SNR, Snoring Scale; SOB, Sleep short of breath or headache; ADQ, Sleep adequacy; SOM, Somnolence Scale; SRSD, Self-reported sleep duration; ESS, Epworth Sleepiness Scale; ISI: Insomnia Severity Index.

Supplementary Table 4. Demographic, concurrent health and sleep variables in original, subset and imputed data

	Original (N= 619)	Unimpaired Subset (N=598)	Imputed (N=1237)
**Age (years) (mean (SD))**	62.63 (6.73)	62.50 (6.75)	62.94 (6.52)
**Female (%)**	432 (69.8)	419 (70.1)	864 (69.8)
**White/Caucasian (%)**	521 (84.2)	504 (84.3)	1126 (91.0)
**College (%)**	376 (60.9)	367 (61.6)	761 (61.6)
***APOE* e4 carriers positive (%)**	207 (38.5)	198 (37.9)	447 (38.7)
**WRAT3 Reading (median [IQR])**	107.00 [100.00, 115.00]	108.00 [100.00, 115.00]	109.00 [101.00, 115.00]
**Concurrent health**			
**CES_D Score (median [IQR])**	5.00 [2.00, 10.00]	5.00 [2.00, 10.00]	5.00 [2.00, 9.00]
**SRH**			
**Poor**	6 (1.0)	5 (0.8)	7 (0.6)
**Fair**	45 (7.3)	42 (7.1)	77 (6.3)
**Good**	247 (40.2)	237 (39.9)	475 (38.6)
**Very Good**	254 (41.4)	249 (41.9)	516 (42.0)
**Excellent**	62 (10.1)	61 (10.3)	155 (12.6)
**Num_prescription (median [IQR])**	3.00 [1.00, 5.00]	2.00 [1.00, 5.00]	2.00 [1.00, 5.00]
**Self-rated memory**			
**Major Problems**	75 (12.1)	69 (11.5)	142 (11.5)
**Neutral**	109 (17.6)	102 (17.1)	257 (20.8)
**No Problems**	435 (70.3)	427 (71.4)	836 (67.7)
**BMI**	28.96 [25.10, 33.96]	28.93 [24.97, 33.61]	28.35 [24.71, 32.84]
**Sleep Variables**			
**SPI1 (median [IQR])**	4.83 [4.33, 5.33]	4.83 [4.33, 5.33]	5.00 [4.33, 5.33]
**SDS (median [IQR])**	4.75 [4.00, 5.25]	4.75 [4.00, 5.25]	4.75 [4.25, 5.25]
**ADQ (median [IQR])**	4.00 [3.00, 5.00]	4.00 [3.00, 5.00]	4.50 [3.50, 5.00]
**SOM (median [IQR])**	5.00 [4.33, 5.67]	5.00 [4.33, 5.67]	5.00 [4.33, 5.67]
**SRSR (median [IQR])**	7.00 [6.00, 8.00]	7.00 [6.00, 8.00]	7.00 [6.00, 8.00]
**ESS (median [IQR])**	2.25 [1.88, 2.62]	2.38 [2.00, 2.62]	2.25 [2.00, 2.62]
**ISI (median [IQR])**	4.29 [3.71, 4.71]	4.29 [3.71, 4.71]	4.29 [3.86, 4.71]
**SPI2 (median [IQR])**	4.78 [4.11, 5.11]	4.78 [4.22, 5.11]	4.89 [4.33, 5.22]
**SNR (median [IQR])**	5.00 [4.00, 5.00]	5.00 [4.00, 5.00]	5.00 [4.00, 6.00]
**SOB (median [IQR])**	6.00 [6.00, 6.00]	6.00 [6.00, 6.00]	6.00 [6.00, 6.00]

*College=Education years>=16, CES_D = Center for Epidemiological Studies Depression Scale, SRH = self-reported health, Num_prescription = total number of prescriptions, BMI = Body Mass Index, SPI1=Sleep-Problem Index I, SPI2= Sleep-Problem Index II, SDS=Sleep Disturbance scale, SNR=Snoring Scale, SOB=Sleep short of breath or headache, ADQ=Sleep adequacy, SOM, Somnolence Scale, SRSD, Self-reported sleep duration, ESS=Epworth Sleepiness Scale, ISI = Insomnia Severity Index.

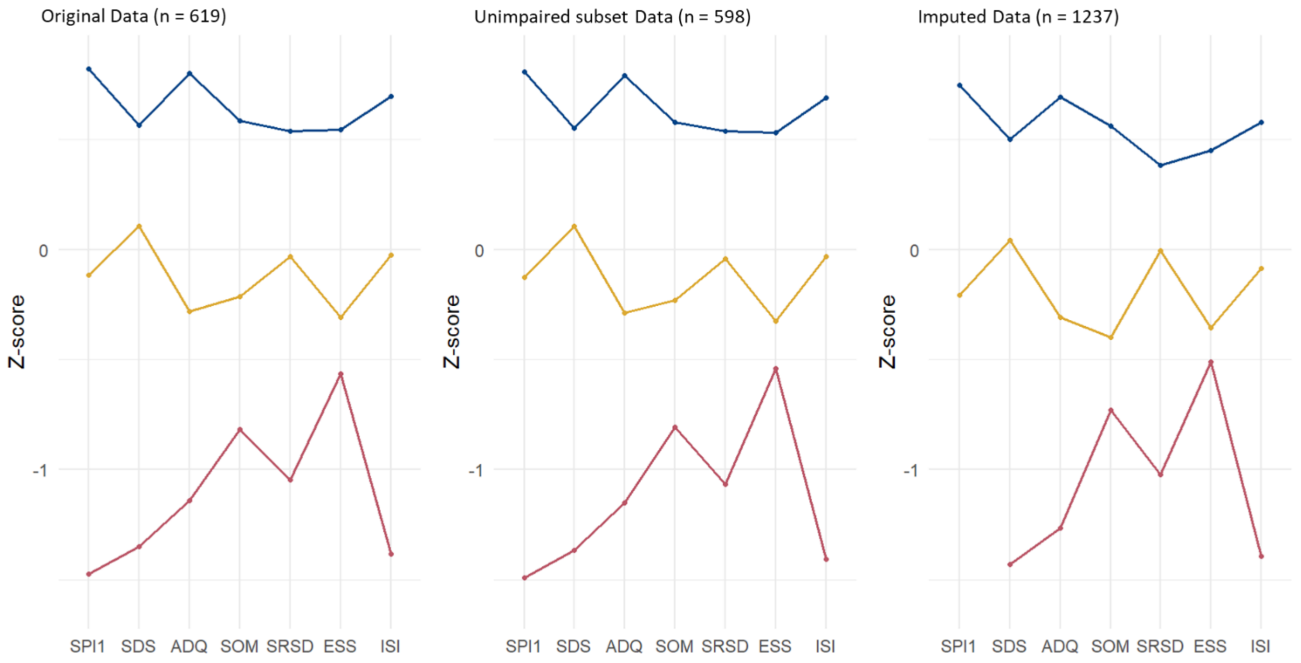

Supplementary Figure 3. Comparison of Kmeans analysis. We visualize the sleep z scores by cluster using Kmeans analysis in three datasets. Original Data is the data used in the primary analysis, Unimpaired Subset Data is the data excluding participants who were MCI at any visits, and Imputed Data is the imputed data. The y axis is the mean z-scores of each sleep variable within each sleep group. Abbreviations: SPI1, Sleep-Problem Index I; SDS, Sleep Disturbance scale; ADQ, Sleep adequacy; SOM, Somnolence Scale; SRSD, Self-reported sleep duration; ESS, Epworth Sleepiness Scale; ISI: Insomnia Severity Index.

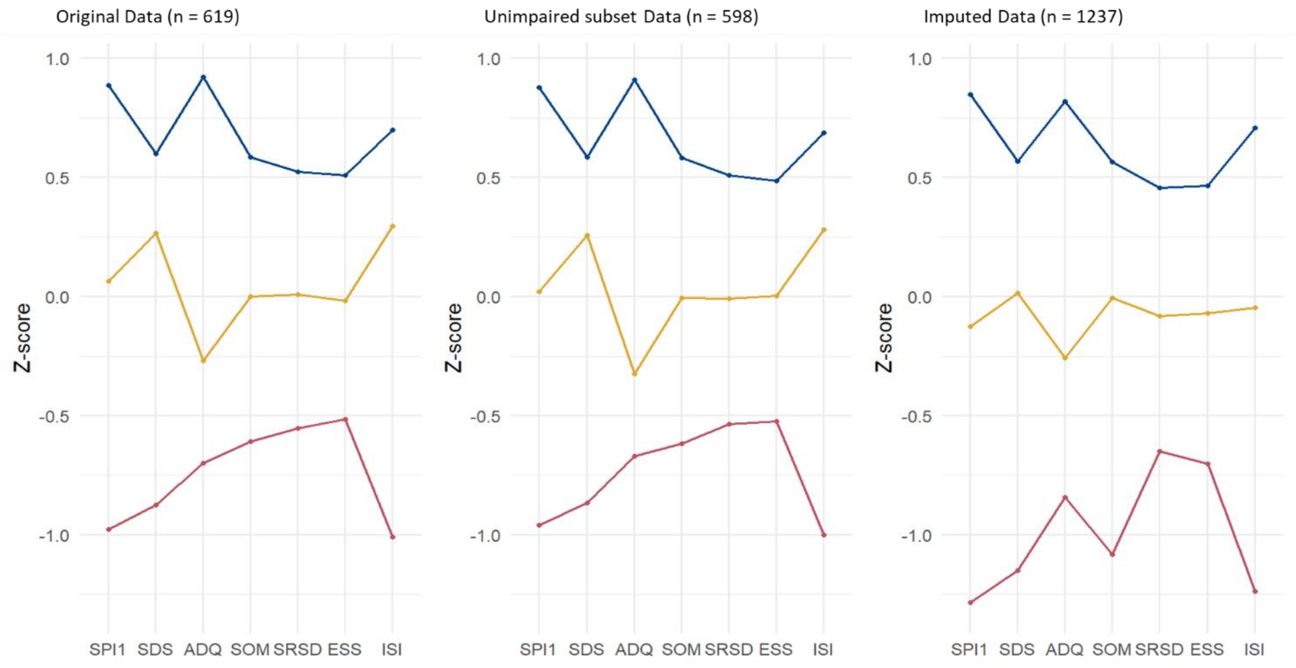

Supplementary Figure 4. Comparison of Latent profile analysis. We visualize the sleep z scores by cluster using Latent profile analysis in three datasets.Original Data is the data used in main analysis, Unimpaired Subset Data is the data excluding participants who were MCI at any visits, and Imputed Data is the imputed data. The y axis is the mean z-scores of each sleep variable within each sleep groups. Abbreviations: SPI1, Sleep-Problem Index I; SDS, Sleep Disturbance scale; ADQ, Sleep adequacy; SOM, Somnolence Scale; SRSD, Self-reported sleep duration; ESS, Epworth Sleepiness Scale; ISI: Insomnia Severity Index.

A **B**

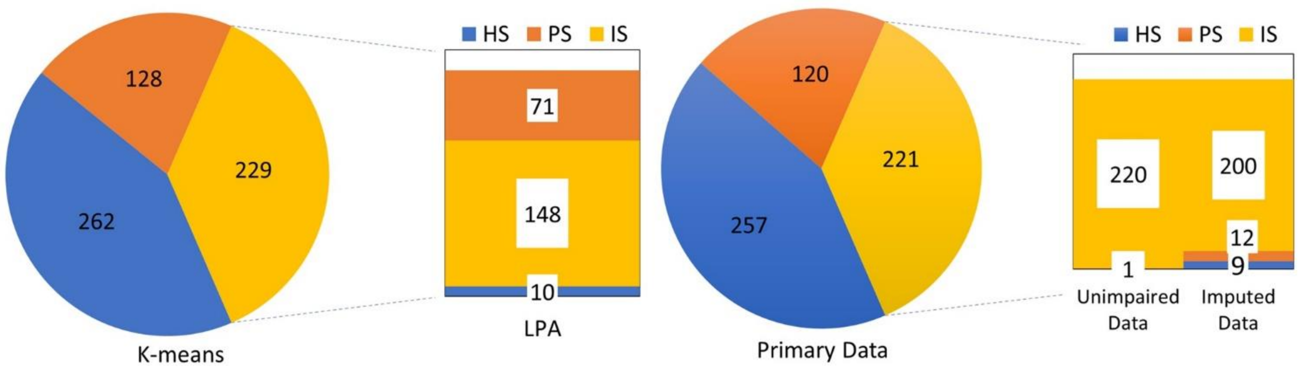

Supplementary Figure 5. The disagreements description between (A) K-means and LPA cluster results, and (B) 598 participants in three datasets. (A) Using LPA, 10 IS classified by Kmeans cluster analysis switch to HS group, 71 IS switch to PS group. (B) In the cluster results among 598 participants of three datasets, 1 IS switch to PS in Unimpaired Subset Data, 9 IS switch to HS and 12 IS switch to PS in Imputed Data.

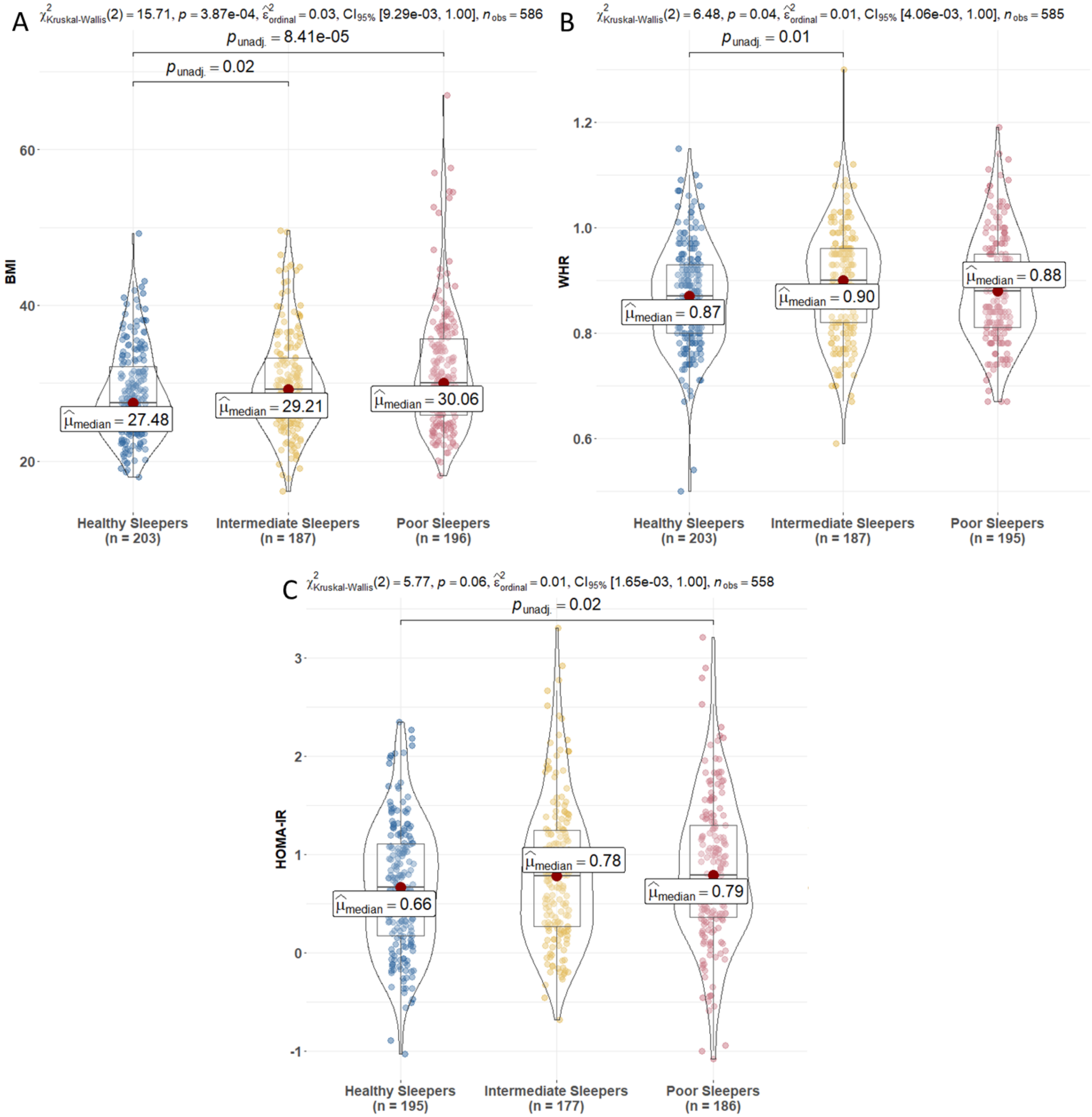

Supplementary Figure 6. Comparison of metabolic biomarkers at sleep baseline among LPA sleep profiles in original dataset. (A) BMI (B) WHR (C) HOMA-IR is calculated by: glucose (mg/dL) * insulin (mIU/mL)/405 (excluding people on insulin therapy). In figure, HOMA-IR is log transformed. **Abbreviations:** BMI, Body mass index; WHR, Waist-to-Hip Ratio; HOMA-IR, Homeostatic Model Assessment of Insulin Resistance. Post hoc significant pairwise group differences at unadjusted P < 0.05 are shown in the figure.

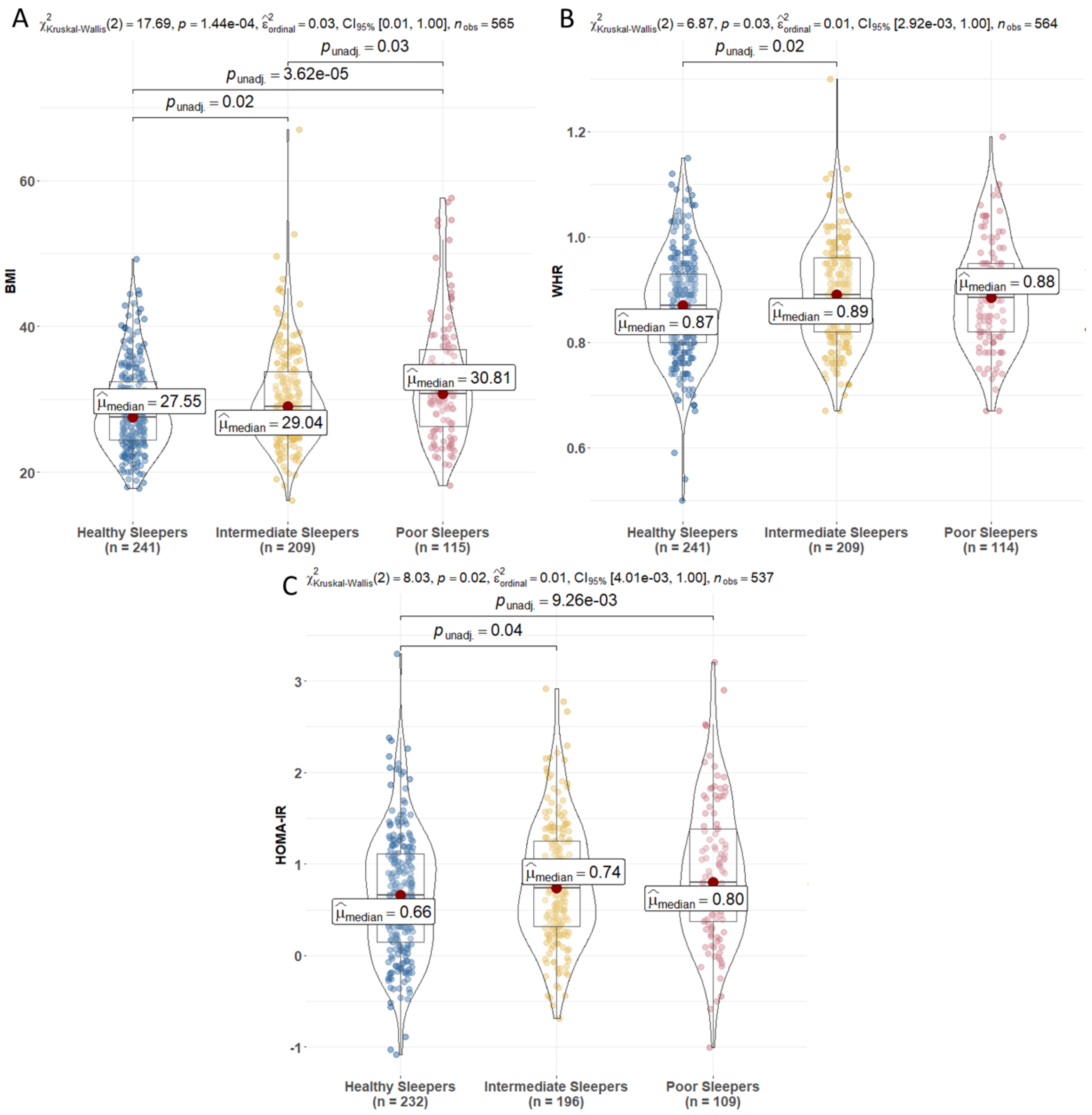

Supplementary Figure 7. Comparison of metabolic biomarkers at sleep baseline among sleep profiles in Unimpaired Subset. (A) BMI (B) WHR (C) HOMA-IR is calculated by: glucose (mg/dL) * insulin (mIU/mL)/405 (excluding people on insulin therapy). In figure, HOMA-IR is log transformed. **Abbreviations:** BMI, Body mass index; WHR, Waist-to-Hip Ratio; HOMA-IR, Homeostatic Model Assessment of Insulin Resistance. Post hoc significant pairwise group differences at unadjusted P < 0.05 are shown in the figure.

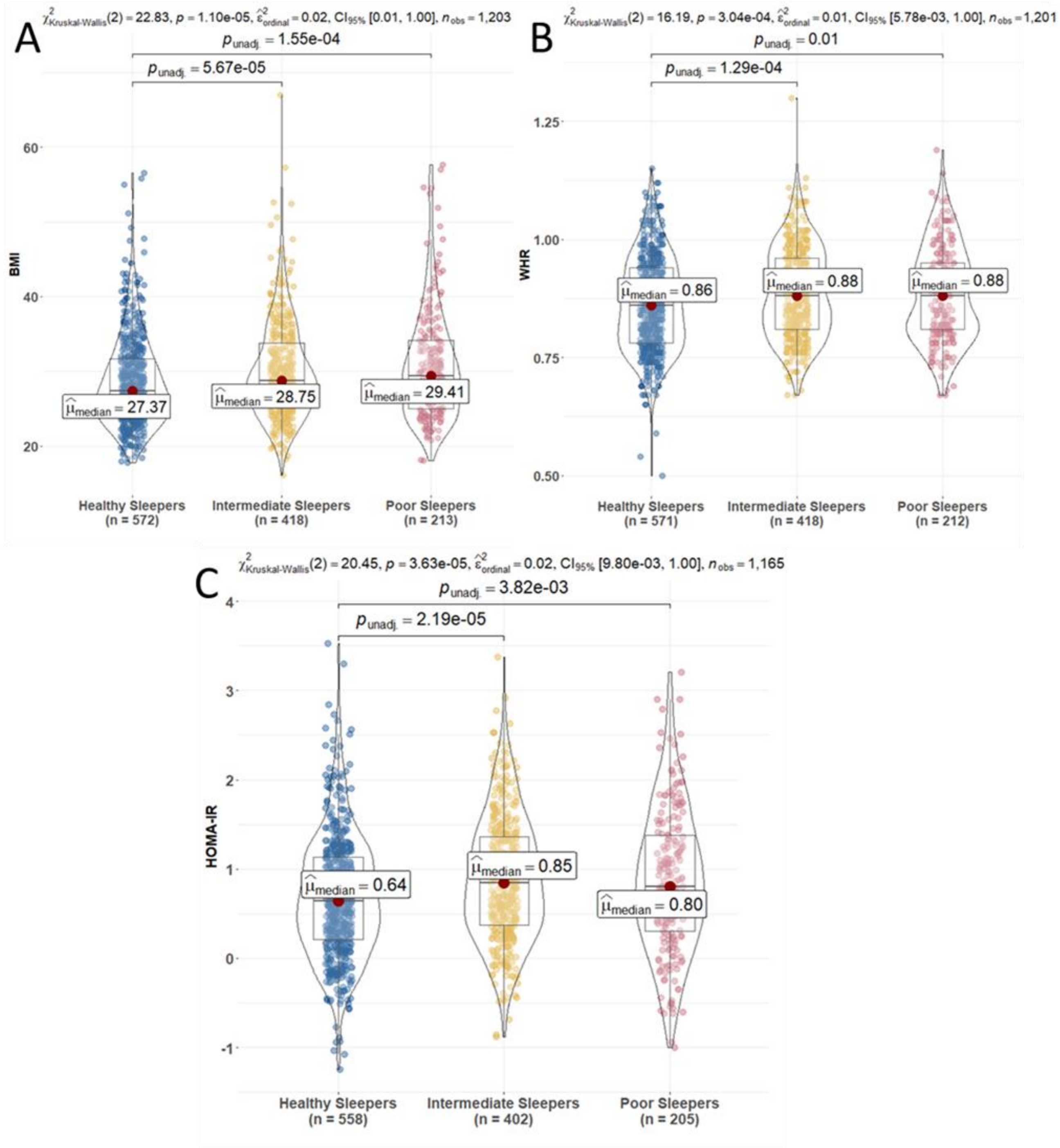

Supplementary Figure 8. Comparison of metabolic biomarkers at sleep baseline among sleep profiles in Imputed Data. (A) BMI (B) WHR (C) HOMA-IR is calculated by: glucose (mg/dL) * insulin (mIU/mL)/405 (excluding people on insulin therapy). In figure, HOMA-IR is log transformed. **Abbreviations:** BMI, Body mass index; WHR, Waist-to-Hip Ratio; HOMA-IR, Homeostatic Model Assessment of Insulin Resistance. Post hoc significant pairwise group differences at unadjusted P < 0.05 are shown in the figure.

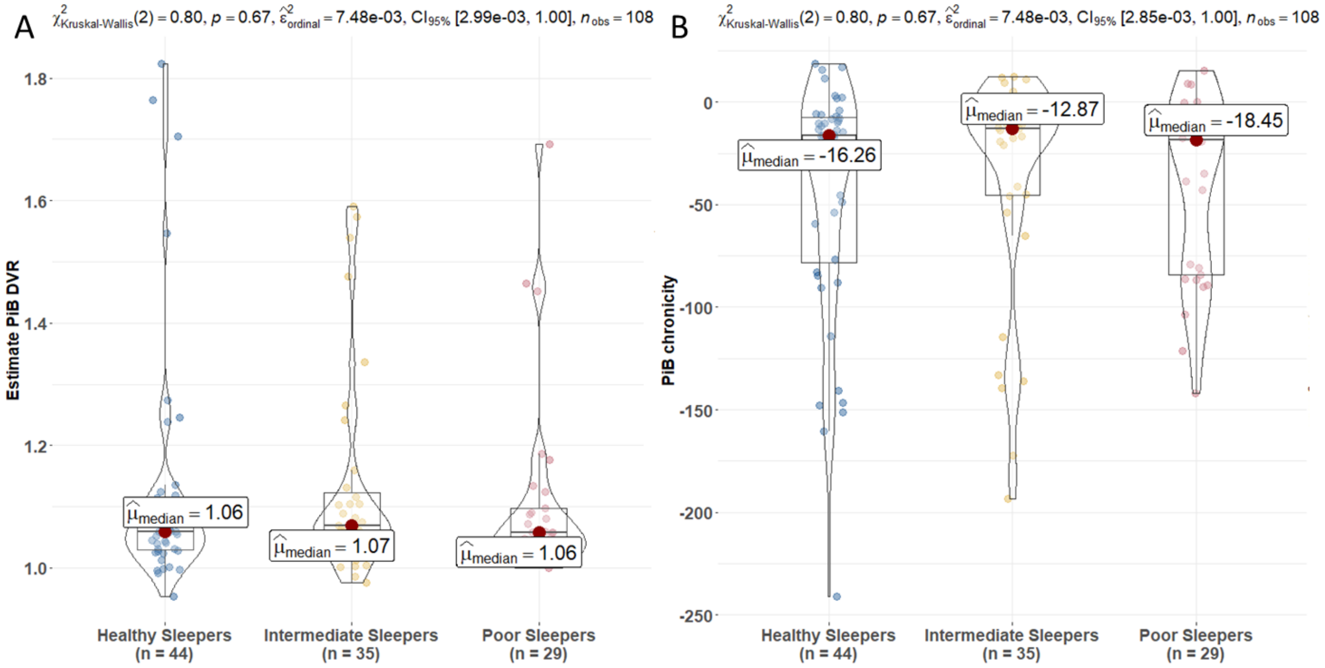

Supplementary Figure 9. Comparison of PET amyloid measures at sleep baseline among LPA sleep profiles in original dataset. (A) Estimates build on the PiB DVR (B) amyloid chronicity described in Koscik, Betthauser et al., 2020. **Abbreviations:** PiB, Pittsburgh Compound B; DVR distribution volume ratio. Post hoc significant pairwise group differences at unadjusted P < 0.05 are shown in the figure.

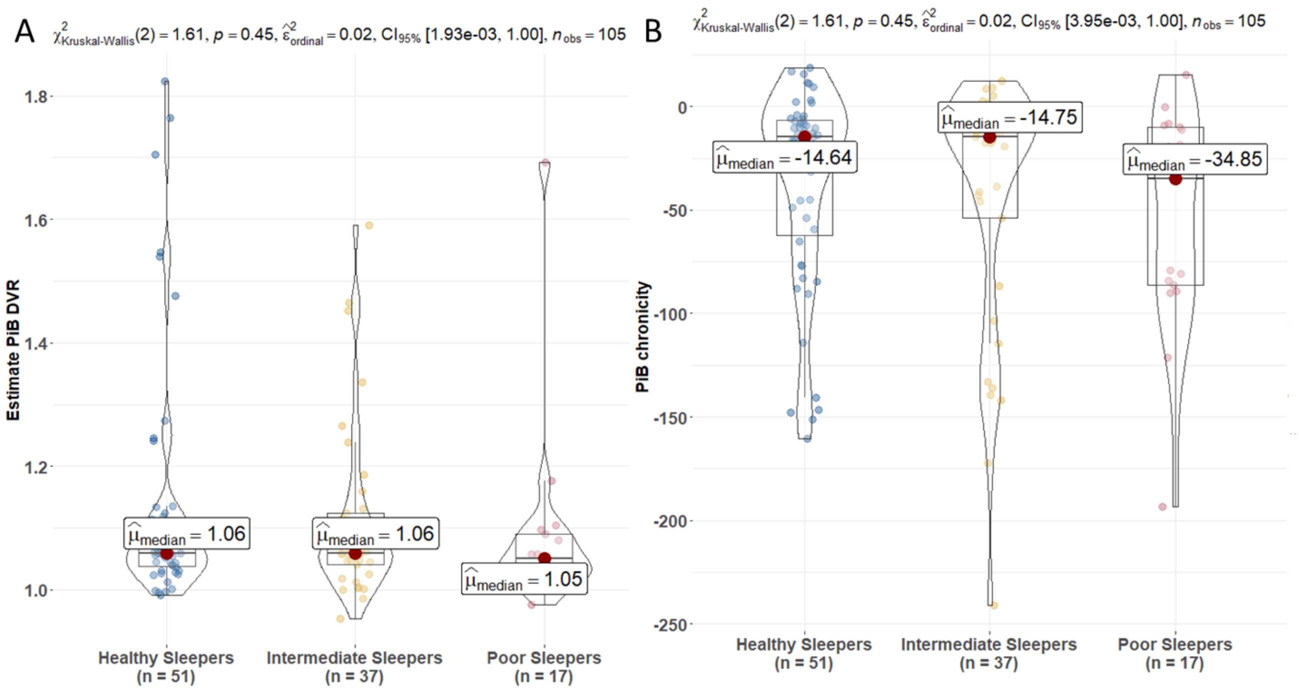

Supplementary Figure 10. Comparison of PET amyloid measures at sleep baseline among sleep profiles in Unimpaired Subset. (A) Estimates build on the PiB DVR (B) amyloid chronicity described in Koscik, Betthauser et al., 2020. **Abbreviations:** PiB, Pittsburgh Compound B; DVR distribution volume ratio. Post hoc significant pairwise group differences at unadjusted P < 0.05 are shown in the figure.

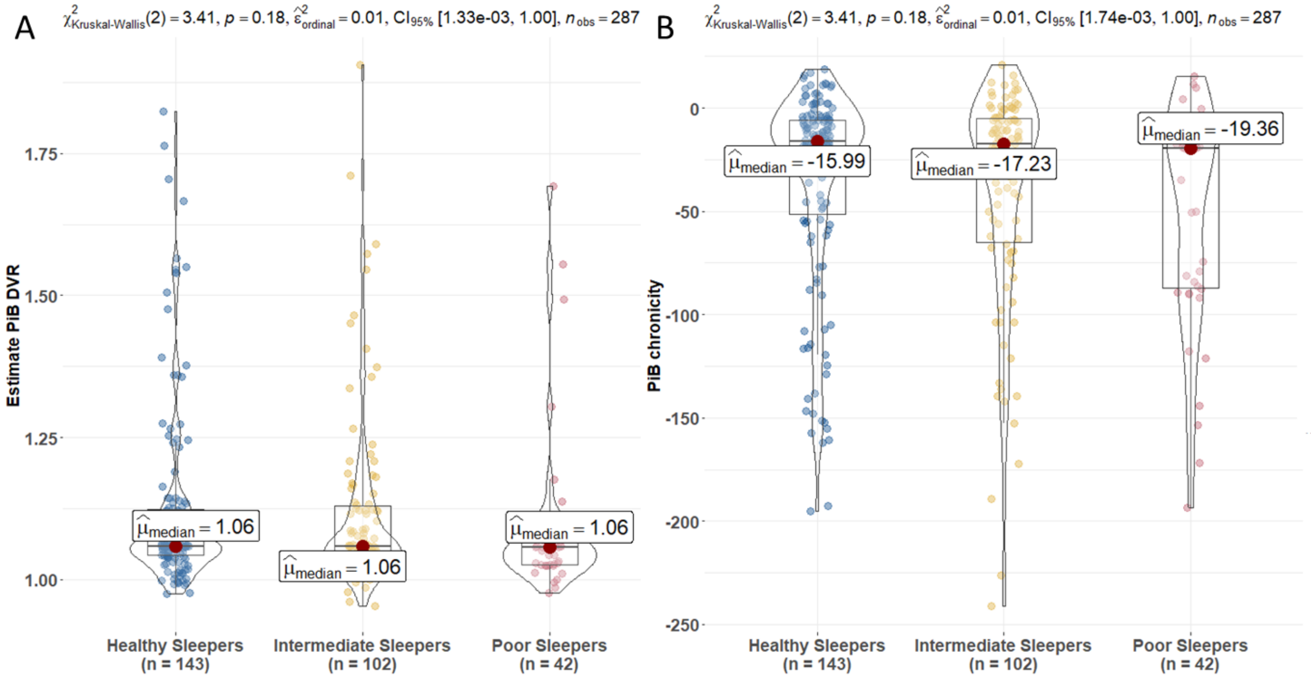

Supplementary Figure 11. Comparison of PET amyloid measures at sleep baseline among sleep profiles in Imputed Data. (A) Estimates build on the PiB DVR (B) amyloid chronicity described in Koscik, Betthauser et al., 2020. **Abbreviations:** PiB, Pittsburgh Compound B; DVR distribution volume ratio. Post hoc significant pairwise group differences at unadjusted P < 0.05 are shown in the figure.

Supplementary Table 5. The associations between sleep groups and PACC3.

**PACC3**	**Model 1**	**Model 2**	**Model 3**	**Model 4**	**Model 5**	**Model 6**	**Model 7**
Predictors	*Estimates(CI)*	*Estimates(CI)*	*Estimates(CI)*	*Estimates(CI)*	*Estimates(CI)*	*Estimates(CI)*	*Estimates(CI)*
Age	-0.05(-0.06 – -0.04)***	-0.05(-0.06 – -0.04)***	-0.05(-0.06 – -0.04)***	-0.05(-0.06 – -0.04)***	-0.05(-0.06 – -0.04)***	-0.05(-0.06 – -0.04)***	-0.05(-0.06 – -0.04)***
Female	0.4(0.28 – 0.52) ***	0.41(0.29 – 0.52) ***	0.4(0.32 – 0.49) ***	0.41(0.28 – 0.54) ***	0.43(0.30 – 0.56) ***	0.43(0.31 – 0.56) ***	0.45(0.32 – 0.57) ***
WRAT3 Reading	0.02(0.01 – 0.03) ***	0.02(0.02 – 0.03) ***	0.02(0.02 – 0.03) ***	0.02(0.01 – 0.03) ***	0.02(0.01 – 0.03) ***	0.02(0.01 – 0.03) ***	0.02(0.01 – 0.03) ***
College	0.3(0.16 – 0.43) ***	0.27(0.14 – 0.39) ***	0.22(0.13 – 0.31) ***	0.27(0.13 – 0.41) ***	0.33(0.19 – 0.47) ***	0.33(0.18 – 0.47) ***	0.32(0.17 – 0.46) ***
Intermediate Sleepers	-0.23(-0.36 – -0.11)***	-0.21(-0.34 – -0.09)**	-0.14(-0.22 – -0.05)**	-0.24(-0.38 – -0.11)***	-0.24(-0.38 – -0.11)***	-0.24(-0.37 – -0.10)**	-0.2(-0.35 – -0.06)**
Poor Sleepers	-0.31(-0.46 – -0.16)***	-0.29(-0.44 – -0.15)***	-0.26(-0.37 – -0.15)***	-0.3(-0.47 – -0.14)***	-0.34(-0.51 – -0.17)***	-0.33(-0.50 – -0.16)***	-0.34(-0.49 – -0.19)***
practice	0.17(0.12 – 0.23)***	0.17(0.11 – 0.22)***	0.18(0.14 – 0.23)***	0.18(0.11 – 0.24) ***	0.16(0.07 – 0.26) **	0.16(0.06 – 0.25) **	0.15(0.06 – 0.25) **
APOE-e4 carriers positive						-0.11(-0.24 – 0.01)	-0.12(-0.25 – -0.00)*
AICc	1176.44	1070.59	2390.99	1035.19	1022.41	1021.23	1019.85
Observations	567	546	1176	503	495	495	495
R^2 / R^2 adjusted	0.372 / 0.364	0.370 / 0.362	0.330 / 0.326	0.372 / 0.363	0.351 / 0.342	0.355 / 0.345	0.357 / 0.346

Note: College=Education years>=16. AICc = corrected Akaike Information Criterion. P value *0.05, **0.01, *** 0.001.

Model 1: The original data in the main manuscript. Model 2: The unimpaired subset data. Model 3: The imputed data. Model 4: Exclude stroke, epilepsy/seizures, multiple sclerosis (MS) and parkinsons. Model 5: Data with completed *APOE* data. Model 6: That same data in Model 5 + covariates *APOE*. Model 7: The same data and covariates using the sleep clusters derived by Latent profile analysis.

Supplementary Table 6. The associations between sleep groups and Executive function.

**EF**	**Model 1**	**Model 2**	**Model 3**	**Model 4**	**Model 5**	**Model 6**	**Model 7**
Predictors	*Estimates(CI)*	*Estimates(CI)*	*Estimates(CI)*	*Estimates(CI)*	*Estimates(CI)*	*Estimates(CI)*	*Estimates(CI)*
Age	-0.06(-0.07 – -0.05)***	-0.06(-0.07 – -0.05)***	-0.06(-0.07 – -0.06)***	-0.06(-0.07 – -0.05)***	-0.06(-0.07 – -0.05)***	-0.06(-0.07 – -0.05)***	-0.06(-0.07 – -0.05)***
Female	0.27(0.13 – 0.41) ***	0.26(0.12 – 0.39) ***	0.27(0.18 – 0.37) ***	0.25(0.11 – 0.40)**	0.25(0.10 – 0.41)**	0.26(0.11 – 0.41)**	0.28(0.13 – 0.43) ***
WRAT3 Reading	0.02(0.02 – 0.03) ***	0.02(0.02 – 0.03) ***	0.02(0.02 – 0.03) ***	0.03(0.02 – 0.03) ***	0.02(0.01 – 0.03) ***	0.02(0.01 – 0.03) ***	0.02(0.01 – 0.03) ***
College	0.13(-0.02 – 0.28)	0.09(-0.05 – 0.24)	0.05(-0.04 – 0.15)	0.15(-0.01 – 0.30)	0.19(0.02 – 0.36)*	0.19(0.02 – 0.35)*	0.18(0.01 – 0.34)*
Intermediate Sleepers	-0.1(-0.25 – 0.04)	-0.07(-0.21 – 0.07)	-0.13(-0.23 – -0.04)**	-0.09(-0.24 – 0.06)	-0.11(-0.26 – 0.04)	-0.1(-0.26 – 0.05)	-0.03(-0.20 – 0.14)
Poor Sleepers	-0.25(-0.43 – -0.07)**	-0.19(-0.36 – -0.02)*	-0.22(-0.35 – -0.10)***	-0.21(-0.39 – -0.02)*	-0.24(-0.43 – -0.04)*	-0.23(-0.42 – -0.03)*	-0.25(-0.42 – -0.07)**
practice	0.1(0.03 – 0.16)**	0.08(0.02 – 0.15)**	0.1(0.05 – 0.16)***	0.09(0.02 – 0.16)*	0.01(-0.10 – 0.12)	0.01(-0.10 – 0.12)	0.01(-0.10 – 0.12)
APOE-e4 carriers positive						-0.1(-0.24 – 0.04)	-0.11(-0.25 – 0.03)
AICc	1233.55	1107.32	2552.89	1104.51	1100.83	1100.98	1097.24
Observations	536	516	1136	485	475	475	475
R^2 / R^2 adjusted	0.293 / 0.284	0.273 / 0.263	0.278 / 0.273	0.292 / 0.281	0.267 / 0.256	0.270 / 0.258	0.276 / 0.264

Note: College=Education years>=16. AICc = corrected Akaike Information Criterion. P value *0.05, **0.01, *** 0.001.

Model 1: The original data in the main manuscript. Model 2: The unimpaired subset data. Model 3: The imputed data. Model 4: Exclude stroke, epilepsy/seizures, multiple sclerosis (MS) and parkinsons. Model 5: Data with completed *APOE* data. Model 6: That same data in Model 5 + covariates *APOE*. Model 7: The same data and covariates using the sleep clusters derived by Latent profile analysis.

Supplementary Table 7. The associations between sleep groups and Immediate Learning.

**Immediate Learning**	**Model 1**	**Model 2**	**Model 3**	**Model 4**	**Model 5**	**Model 6**	**Model 7**
Predictors	*Estimates(CI)*	*Estimates(CI)*	*Estimates(CI)*	*Estimates(CI)*	*Estimates(CI)*	*Estimates(CI)*	*Estimates(CI)*
Age	-0.05(-0.06 – -0.04)***	-0.05(-0.05 – -0.04)***	-0.04(-0.05 – -0.04)***	-0.05(-0.06 – -0.04)***	-0.05(-0.06 – -0.04)***	-0.05(-0.06 – -0.04)***	-0.05(-0.06 – -0.04)***
Female	0.23(0.10 – 0.36) ***	0.25(0.12 – 0.37) ***	0.28(0.19 – 0.37) ***	0.25(0.11 – 0.38) ***	0.27(0.14 – 0.41) ***	0.28(0.15 – 0.42) ***	0.29(0.15 – 0.43) ***
WRAT3 Reading	0.03(0.02 – 0.04) ***	0.03(0.02 – 0.04) ***	0.03(0.02 – 0.03) ***	0.03(0.02 – 0.04) ***	0.03(0.02 – 0.03) ***	0.03(0.02 – 0.03) ***	0.03(0.02 – 0.03) ***
College	0.19(0.05 – 0.33)**	0.17(0.04 – 0.31)*	0.13(0.04 – 0.23)**	0.14(-0.01 – 0.29)	0.22(0.07 – 0.37)**	0.22(0.07 – 0.37)**	0.21(0.06 – 0.36)**
Intermediate Sleepers	-0.2(-0.34 – -0.07)**	-0.18(-0.31 – -0.05)**	-0.12(-0.21 – -0.03)*	-0.22(-0.36 – -0.07)**	-0.22(-0.36 – -0.07)**	-0.2(-0.34 – -0.06)**	-0.18(-0.34 – -0.03)*
Poor Sleepers	-0.23(-0.39 – -0.06)**	-0.2(-0.36 – -0.05)*	-0.19(-0.30 – -0.07)**	-0.24(-0.42 – -0.07)**	-0.3(-0.48 – -0.12)**	-0.29(-0.47 – -0.11)**	-0.28(-0.44 – -0.12)**
practice	0.15(0.09 – 0.20)***	0.14(0.09 – 0.20)***	0.13(0.08 – 0.19) ***	0.16(0.09 – 0.22) ***	0.16(0.07 – 0.26) **	0.16(0.06 – 0.25) **	0.15(0.06 – 0.25) **
APOE-e4 carriers positive						-0.16(-0.29 – -0.02)*	-0.17(-0.30 – -0.04)*
AICc	1264.24	1155.4	2579.41	1108.88	1093.91	1090.49	1091.02
Observatios	571	550	1184	505	498	498	498
R^2 / R^2 adjusted	0.303 / 0.294	0.307 / 0.298	0.242 / 0.237	0.299 / 0.289	0.291 / 0.281	0.299 / 0.288	0.298 / 0.287

Note: College=Education years>=16. AICc = corrected Akaike Information Criterion. P value *0.05, **0.01, *** 0.001.

Model 1: The original data in the main manuscript. Model 2: The unimpaired subset data. Model 3: The imputed data. Model 4: Exclude stroke, epilepsy/seizures, multiple sclerosis (MS) and parkinsons. Model 5: Data with completed *APOE* data. Model 6: That same data in Model 5 + covariates *APOE*. Model 7: The same data and covariates using the sleep clusters derived by Latent profile analysis.

Supplementary Table 8. The associations between sleep groups and Delayed Recall.

	Model 1	Model 2	Model 3	Model 4	Model 5	Model 6	Model 7
Predictors	*Estimates(CI)*	*Estimates(CI)*	*Estimates(CI)*	*Estimates(CI)*	*Estimates(CI)*	*Estimates(CI)*	*Estimates(CI)*
Age	-0.04(-0.05 – -0.03)***	-0.04(-0.05 – -0.03)***	-0.04(-0.04 – -0.03)***	-0.04(-0.05 – -0.03)***	-0.04(-0.05 – -0.03)***	-0.04(-0.05 – -0.03)***	-0.04(-0.05 – -0.03)***
Female	0.19(0.06 – 0.32)**	0.2(0.08 – 0.32)**	0.25(0.16 – 0.34) ***	0.18(0.05 – 0.32)**	0.22(0.08 – 0.35) **	0.23(0.09 – 0.36)**	0.23(0.09 – 0.37) **
WRAT3 Reading	0.03(0.02 – 0.04) ***	0.03(0.02 – 0.04) ***	0.03(0.02 – 0.03) ***	0.03(0.02 – 0.04)***	0.03(0.02 – 0.04) ***	0.03(0.02 – 0.04) ***	0.03(0.02 – 0.04) ***
College	0.16(0.02 – 0.31)*	0.15(0.01 – 0.28)*	0.12(0.03 – 0.22)*	0.1(-0.05 – 0.25)	0.19(0.04 – 0.34) **	0.19(0.04 – 0.34)*	0.18(0.03 – 0.33)*
Intermediate Sleepers	-0.24(-0.37 – -0.10)**	-0.22(-0.35 – -0.09)**	-0.14(-0.23 – -0.05)**	-0.24(-0.38 – -0.10)**	-0.25(-0.39 – -0.11)**	-0.24(-0.38 – -0.09)**	-0.24(-0.40 – -0.09)**
Poor Sleepers	-0.24(-0.41 – -0.07)**	-0.22(-0.38 – -0.06)**	-0.18(-0.29 – -0.06)**	-0.24(-0.42 – -0.07)**	-0.31(-0.49 – -0.13)**	-0.29(-0.47 – -0.11)**	-0.29(-0.45 – -0.13)***
practice	0.18(0.12 – 0.24) ***	0.18(0.12 – 0.23) ***	0.18(0.13 – 0.23) ***	0.2(0.13 – 0.27) ***	0.21(0.11 – 0.31) ***	0.2(0.10 – 0.30) ***	0.19(0.10 – 0.29) ***
APOE-e4 carriers positive						-0.16(-0.29 – -0.03)*	-0.17(-0.30 – -0.04)*
AICc	1273.17	1131.3	2548.9	1105.92	1084.99	1081.27	1081.28
Observations	569	548	1181	504	496	496	496
R^2 / R^2 adjusted	0.295 / 0.287	0.311 / 0.303	0.241 / 0.237	0.301 / 0.291	0.276 / 0.266	0.285 / 0.273	0.285 / 0.273

Note: College=Education years>=16. AICc = corrected Akaike Information Criterion. P value *0.05, **0.01, *** 0.001.
Model 1: The original data in the main manuscript. Model 2: The unimpaired subset data. Model 3: The imputed data. Model 4: Exclude stroke, epilepsy/seizures, multiple sclerosis (MS) and parkinsons. Model 5: Data with completed *APOE* data. Model 6: That same data in Model 5 + covariates *APOE*. Model 7: The same data and covariates using the sleep clusters derived by Latent profile analysis.

Supplementary Table 9. The associations between sleep groups and Working memory.

	Model 1	Model 2	Model 3	Model 4	Model 5	Model 6	Model 7
Predictors	*Estimates(CI)*	*Estimates(CI)*	*Estimates(CI)*	*Estimates(CI)*	*Estimates(CI)*	*Estimates(CI)*	*Estimates(CI)*
Age	-0.04(-0.05 – -0.03)***	-0.04(-0.05 – -0.03)***	-0.04(-0.05 – -0.03)**	-0.04(-0.05 – -0.03)**	-0.04(-0.06 – -0.03)***	-0.04(-0.06 – -0.03)***	-0.04(-0.06 – -0.03)***
Female	-0.19(-0.35 – -0.04)*	-0.22(-0.38 – -0.06)**	-0.16(-0.27 – -0.05)**	-0.19(-0.35 – -0.02)*	-0.19(-0.36 – -0.02)*	-0.18(-0.35 – -0.01)*	-0.19(-0.35 – -0.02)*
WRAT3 Reading	0.04(0.03 – 0.05) ***	0.04(0.03 – 0.05) ***	0.04(0.04 – 0.05) ***	0.05(0.04 – 0.06)***	0.05(0.04 – 0.06) ***	0.05(0.04 – 0.06) ***	0.05(0.04 – 0.06) ***
College	0.05(-0.12 – 0.23)	0.04(-0.13 – 0.22)	0.03(-0.08 – 0.15)	0.03(-0.15 – 0.21)	0.04(-0.15 – 0.22)	0.03(-0.15 – 0.22)	0.04(-0.15 – 0.22)
Intermediate Sleepers	0.07(-0.10 – 0.23)	0.08(-0.09 – 0.24)	-0.01(-0.13 – 0.10)	0.05(-0.12 – 0.23)	0.04(-0.13 – 0.21)	0.05(-0.12 – 0.23)	0.04(-0.15 – 0.23)
Poor Sleepers	0.15(-0.05 – 0.35)	0.14(-0.06 – 0.35)	0.06(-0.09 – 0.20)	0.19(-0.02 – 0.40)	0.19(-0.03 – 0.41)	0.2(-0.02 – 0.42)	0.16(-0.03 – 0.36)
practice	0.15(0.08 – 0.23)***	0.16(0.09 – 0.24)***	0.14(0.08 – 0.21)***	0.15(0.07 – 0.23)***	0.07(-0.05 – 0.19)	0.06(-0.06 – 0.18)	0.06(-0.06 – 0.18)
APOE-e4 carriers positive						-0.14(-0.30 – 0.02)	-0.13(-0.29 – 0.03)
AICc	1433.99	1383.43	3025.81	1309.67	1281.02	1280.18	1280.28
Observations	557	537	1171	504	494	494	494
R^2 / R^2 adjusted	0.247 / 0.238	0.245 / 0.235	0.200 / 0.195	0.231 / 0.220	0.239 / 0.228	0.244 / 0.231	0.243 / 0.231

Note: College=Education years>=16. AICc = corrected Akaike Information Criterion. P value *0.05, **0.01, *** 0.001.

Model 1: The original data in the main manuscript. Model 2: The unimpaired subset data. Model 3: The imputed data. Model 4: Exclude stroke, epilepsy/seizures, multiple sclerosis (MS) and parkinsons. Model 5: Data with completed *APOE* data. Model 6: That same data in Model 5 + covariates *APOE*. Model 7: The same data and covariates using the sleep clusters derived by Latent profile analysis.

Supplementary Table 10. Association of self-reported sleep characteristics with amyloid load (PiB DVR)

	Sprecher et al. (N=98)				Replication (N=220)			
	**B**	**SE**	**t**	**p**	**B**	**SE**	**t**	**p**
**Sleep adequacy**	−0.002	0.001	−2.503	**0.014**	-0.001	0.0006	-1.859	0.064
**Somnolence**	0.003	0.001	2.171	**0.033**	0.0006	0.0007	0.883	0.379
**Sleep problems index I**	0.003	0.002	1.973	0.052	0.001	0.0009	1.085	0.279
**Sleep problems index II**	0.003	0.002	1.894	0.061	0.0005	0.0009	0.574	0.567
**Sleep short of breath or headache**	0.000	0.001	0.194	0.847	-0.00004	0.0009	-0.042	0.967
**Snoring**	0.000	0.001	0.261	0.795	-0.00003	0.0004	-0.062	0.950
**Sleep Disturbance**	0.001	0.001	0.692	0.491	-0.0004	0.0007	-0.532	0.595
**ESS**	-0.001	0.006	-0.103	0.918	-0.002	0.004	-0.481	0.631
**Sleep Hours**	-0.022	0.019	-1.175	0.243	-0.014	0.012	-1.171	0.242

All models were adjusted for age, sex, *APOE-e4*, family history of Alzheimer's disease, and body mass index. Bold fonts indicate statistically significant P values.
